# Effects of supplemental tannic acid on growth performance, gut health, microbiota, and fat accumulation and optimal dosages of tannic acid in broilers

**DOI:** 10.3389/fphys.2022.912797

**Published:** 2022-09-02

**Authors:** Janghan Choi, Sudhir Yadav, Jinquan Wang, Benjamin J. Lorentz, Jeferson M. Lourenco, Todd R. Callaway, Woo Kyun Kim

**Affiliations:** ^1^ Department of Poultry Science, University of Georgia, Athens, GA, United States; ^2^ Department of Animal and Dairy Science, University of Georgia, Athens, GA, United States

**Keywords:** tannic acid, gut health, apparent ileal digestibility, fat accumulation, microbiota, volatile fatty acids, chickens

## Abstract

This study was conducted to investigate the effects of different dosages of tannic acid (TA) on growth performance, nutrient digestibility, gut health, immune system, oxidative status, microbial composition, volatile fatty acids (VFA), bone mineral density, and fat digestion and accumulation in broilers and to find optimal dosages of TA for efficient growth and gut health in broilers. A total of 320 male Cobb500 broilers were randomly distributed to 4 treatments with 8 replicates including 1) tannic acid 0 (TA0): basal diet without TA; 2) tannic acid 0.5 (TA0.5): basal diet with 0.5 g/kg TA; 3) tannic acid 1.5 (TA1.5); and 4) tannic acid 2.5 (TA2.5). Supplemental TA at levels greater than 972 mg/kg tended to reduce BW on D 21 (*p* = 0.05). The TA2.5 had significantly lower apparent ileal digestibility (AID) of crude protein compared to the TA0 group. The AID of ether extract tended to be reduced by TA at levels greater than 525 mg/kg (*p* = 0.08). The jejunal lipase activities tended to be reduced by TA at levels less than 595.3 mg/kg (*p* = 0.09). TA linearly decreased goblet cell density in the crypts of the jejunum (*p* < 0.05) and reduced mRNA expression of mucin two at levels less than 784.9 mg/kg and zonula occludens two at levels less than 892.6 mg/kg (*p* < 0.05). The TA0.5 group had higher activities of liver superoxide dismutase compared to the TA0 group (*p* < 0.05). Bone mineral density and contents tended to be linearly decreased by TA (*p* = 0.05), and the ratio of lean to fat was linearly decreased (*p* < 0.01). Total cecal VFA production tended to be linearly reduced by TA at levels greater than 850.9 mg/kg (*p* = 0.07). Supplemental TA tended to increase the relative abundance of the phylum Bacteroidetes (*p* = 0.1) and decrease the relative abundance of the phylum Proteobacteria (*p* = 0.1). The relative abundance of the family Rikenellaceae was the lowest at 500 mg/kg TA, and the relative abundance of the family Bacillaceae was the highest at 1,045 mg/kg TA. Collectively, these results indicate that the optimum level of supplemental TA would range between 500 and 900 mg/kg; this range of TA supplementation would improve gut health without negatively affecting growth performance in broilers under antibiotic-free conditions.

## Introduction

There are many challenges in poultry production such as pathogenic bacteria infection, parasitic infection, heat stress, etc. That can impair production efficiency (Choi and Kim, 2020). To cope with these issues, antibiotics as growth promoters (AGP) have been supplemented in poultry diets (Pedroso et al., 2006). However, the continuous use of AGP has led to an increase in the spread of resistant bacteria and their resistant genes (Zhang et al., 2015). Due to the public demand to reduce the use of AGP, many countries have banned or restricted the use of AGP in poultry (Luise et al., 2022). Many bioactive compounds including essential oils (Yang et al., 2021), plant extracts (Mogire et al., 2021; Yadav et al., 2022), organic acids (Pham et al., 2020), exogenous enzymes (Lu et al., 2020), probiotics (Adhikari and Kim, 2017), and prebiotics (Teng and Kim, 2018) were studied to replace AGP in poultry production. Potential AGP replacements should be cost-effective and be able to improve growth performance *via* exhibiting antimicrobial, antioxidative, and anti-inflammatory effects in chickens (Yang et al., 2015).

Tannins, plant secondary metabolites, are polyphenol compounds that can precipitate proteins (Sarni-Manchado et al., 1999). Tannins can be mainly categorized into condensed tannins and hydrolysable tannins (Huang et al., 2018). Hydrolysable tannins may have higher bioavailability compared to condensed tannins because hydrolysable tannins can be hydrolyzed and absorbed in the gastrointestinal tract of chickens (Choi and Kim, 2020). Tannic acid (TA), a standard of hydrolysable tannins, is considered as a potential AGP alternative mainly due to their strong antimicrobial, antioxidative, and anti-inflammatory effects, while the high dosages of TA can exhibit anti-nutritional effects in chickens (Choi and Kim, 2020). Supplementation of TA reduced oocyst shedding of *E. maxima* and improved nutrient utilization and gut barrier integrity in broilers infected with *E. maxima* (Choi et al., 2022). A previous study by Ebrahim et al. (2015) also reported that supplemental TA was beneficial to enhance the fatty acid profile of breast muscle of broilers subjected to heat stress. Along with the investigation of beneficial effects of supplemental TA in broilers under challenging conditions, the effects of supplemental TA on growth performance, nutrient digestibility, and gut health in broilers without any challenging conditions should be investigated to apply supplemental TA in poultry production. It is important to identify the optimal dosage range of supplemental TA without anti-nutritional effects in broilers under healthy conditions because TA can be beneficial as well as detrimental depending on its supplemental dosage. Moreover, supplemental TA potentially can ameliorate oxidative stress and inflammation caused by antinutritional factors in soybean meal and fast growth rate in modern broilers (Cheng et al., 2019; Choi et al., 2020a). Therefore, the hypothesis of the study was that supplemental TA at optimal dosages would enhance growth performance, nutrient digestibility, gut health, immune system, oxidative status, microbiota, and body composition in broilers. The objectives of the study were 1) to investigate the effects of different dosages of supplemental TA on growth performance, nutrient digestibility, gut health, immune system, oxidative status, microbial composition and activities, bone mineral density, and fat digestion and accumulation in broilers; and 2) to find optimal dosages of TA for efficient growth and gut health in broilers reared D 0 to 21.

## Materials and methods

### Experimental design, diets, management, and growth performance

The current study was approved by the Institutional Animal Care and Use Committee at the University of Georgia, Athens, GA. A total of 320 1-day old male Cobb500 broilers were randomly allocated to 4 treatments with 8 replicates per treatment of 10 birds per pen in a completely randomized design. The basal diets for the starter (D 0 to 14) and grower (D 14 to 21) phase were formulated to meet or exceed energy and nutrient requirements based on Cobb Broiler Management Guide (Diaz Carrasco et al., 2018) as shown in Table 1. Four dietary treatments included 1) basal diet without TA (ACS reagent; Sigma–Aldrich, St Louis, MO, United States; TA0); 2) basal diet with 0.5 g/kg TA (TA0.5); 3) basal diet with 1.5 g/kg TA (TA1.5); and 4) basal diet with 2.5 g/kg TA (TA2.5). During the whole experimental period, birds were allowed to eat and drink freely (D 0 to 21), and temperature and light were controlled in accordance with Cobb Broiler Management Guide (Diaz Carrasco et al., 2018). On D 7, 14, and 21, body weight (BW) and feed disappearance were recorded to calculate average daily gain (ADG), average daily feed intake (ADFI), and feed conversion ratio (FCR).

**TABLE 1:** Ingredients and nutrient compositions of basal diets (As-fed basis).

Items	D 0 to 14	D 14 to 21
Ingredients		
Corn	650.97	683.74
Soybean meal (480 g crude protein/kg)	295.05	260.05
Defluorinated Phosphate	16.62	14.96
Filler^1^	10	10
Soybean oil	8.27	12.91
limestone	6.45	6.24
DL-Methionine 99%	3.18	2.94
L-Lysine HCl 78%	3.01	2.85
Vitamin Premix^2^	2.5	2.5
Common Salt	1.45	1.66
L-threonine	1.20	0.86
Mineral Premix^3^	0.8	0.8
Monensin sodium^4^	0.5	0.5
Total	1,000	1,000
Calculated energy and nutrient value, %		
Metabolizable energy, Kcal/kg	3,000	3,060
Crude protein	20.59	19.13
SID^5^ Methionine	0.61	0.57
SID Total sulfur amino acids	0.88	0.82
SID Lysine	1.17	1.07
SID Threonine	0.78	0.7
Total calcium	0.87	0.8
Available phosphate	0.44	0.4

1 Sand and tannic acid were added to obtain desired tannic acid dosages in the feed as follows: Tannic acid 0 (TA0): sand 10 g/kg + tannic acid 0 g/kg; Tannic acid 0.5 (TA0.5): sand 9.5 g/kg + tannic acid 0.5 g/kg; Tannic acid 1.5 (TA1.5): sand 8.5 g/kg + tannic acid 1.5 g/kg; Tannic acid 2.5 (TA2.5): sand 7.5 g/kg + tannic acid 2.5 g/kg; Tannic acid was purchased from Sigma–Aldrich (St Louis, MO, United States).

2 Vitamin mix provided the following in mg/100 g diet: thiamine-HCl, 1.5; riboflavin, 1.5; nicotinic acid amide, 15; folic acid, 7.5; pyridoxine-HCl, 1.2; D-biotin 3, 1.5; vitamin B-12 (source concentrations, 0.1%), 2; choline-Cl, 3; D-calcium pantothenate, 4; menadione sodium bisulfite, 1.98; α-tocopherol acetate (source 500,000 IU/g), 22.8; cholecalciferol (source 5,000,000 IU/g), 0.09; retinyl palmitate (source 500,000 IU/g), 2.8; ethoxyquin, 13.34; I-inositol, 2.5; and dextrose, 762.2.

3 Mineral mix provided the following in g/100 g diet: Ca(H_2_PO_4_)_2_ · H_2_O, 3.62; CaCO_3_, 1.48; KH_2_PO_4_, 1.00; Na_2_SeO_4_, 0.0002; MnSO_4_ · H_2_O, 0.035; FeSO_4_ · 7H_2_O, 0.05; MgSO_4_ · 7H_2_O, 0.62; KIO_3_, 0.001; NaCl, 0.60; CuSO_4_ · 5H_2_O, 0.008; ZnCO_3_, 0.015; CoCl_2_ · 6H_2_O, 0.00032; NaMoO_4_ · 2H_2_O, 0.0011; KCl, 0.10; and dextrose, 0.40.

4 Coban 90, Elanco Animal Health, Greenfield, IN, United States

5 SID, tandardized ileal digestible.

### Sample collection

On D 21, birds were euthanized *via* cervical dislocation. Afterwards, blood samples were collected in heparin free vacutainers (Grainer Bio-One, Kremsmuenster, Austria) from the hearts. After allowed to clot for 1 h at room temperature, the samples were centrifuge at 1,000 × g for 10 min to recover serum. The serum samples were stored at −80°C for further analyses. The ceca and liver samples and a 10 cm segment of the mid-jejunum samples were collected and snap-frozen using liquid nitrogen and stored at −80°C for future analyses. A 2 cm of segment of the mid-jejunum and mid-ceca were collected and fixed in a 10% formaldehyde solution. All tissue samples except ceca samples were washed with PBS to remove blood and digesta. From 10 cm below Meckel’s diverticulum to the upper 10 cm of the ileo-cecal-colic junction, ileal digesta samples were collected.

### Apparent ileal digestibility of dry matter, organic matter, ash, crude protein, and ether extract

According to Short et al. (1996), the concentrations of titanium dioxide were measured in oven-dried samples (0.3 g for ileal digesta and 0.5 g for the feed samples). The crude protein (CP) was determined by nitrogen combustion analyses according to AOAC international (2000) analytical method 990.03. The ether extract (EE) content was analyzed according to AOAC international (2000) analytical method 942.05. Apparent ileal digestibility (AID) of dry matter (DM), organic matter (OM), ash, CP, and EE were calculated according to Lin and Olukosi (2021).

### Jejunal and cecal morphology and goblet cell counting

The alcian blue/period acid-Schiff (AB/PAS) staining was performed to measure villus height (VH), crypts depth (CD), and the VH:CD ratio and to count the number of goblet cells in VH and CD in the jejunum and ceca (only crypts) samples. Samples were fixed in 10% neutral-buffered formalin for further analyses. Afterwards, the jejunal and cecal samples were cut cross-sectionally to 0.5 cm and put in cassettes. The sections were stained with alcian blue for 15 min and washed with distilled water. The samples were treated with periodic acid for 5 min and washed with distilled water. Subsequently, the samples were stained with Schiff’s reagents for 10 min and washed with distilled water. Finally, the samples were counterstained in haematoxylin for 1 min and washed and dehydrated. The stained sections were pictured with a microscope (BZ-X810; Keyence, Osaka, Japan). Images (×4) were analyzed using ImageJ (National Institutes of Health, Bethesda, MD, United States).

### Activities of brush border digestive enzymes and serum alkaline phosphatase in the mid-jejunum

Approximately 100 mg of the whole mid-jejunum samples with 2 ml PBS were homogenized using a beads beater (Biospec Products, Bartlesville, OK, United States). The supernatants of homogenized samples after the centrifugation at 4°C and 12,000 × g for 15 min were collected to analyze their protein contents using Pierce BCA Protein Assay Kits (Thermo Fisher Scientific, MA, United States) with a 10-time sample dilution. By using the supernatants, activities of maltase and sucrase were determined according to Fan et al. (2004). Activities of alanine-aminopeptidase (APN) were analyzed according to Maroux et al. (1973). Activities of serum alkaline phosphatase and intestinal alkaline phosphatase (IAP) were determined according to the method from Lackeyram et al. (2010). Lipase activities were determined according to Elgharbawy et al. (2018). The activities of digestive enzymes were expressed as values per mg protein.

### RNA extraction and real-time polymerase chain reaction analysis

Approximately 100 mg of the whole mid-jejunum and liver samples were homogenized in QIAzol lysis reagents (Qiagen, Valencia, CA, United States), and RNAs were extracted according to the manufacturer’s procedure. A NanoDrop 2000 spectrophotometer (Thermo Fisher Scientific) was used to determine RNA quantity and purity. One microgram of RNA was utilized to make the first-strand cDNA using high-capacity cDNA synthesis kits (Applied Biosystems, Foster City, CA, United States). The primers used in the study were presented in Table 2. Real-time PCR was performed using SYBR Green Master Mix with a Step One thermocycler (Applied Biosystem). The final PCR volume (10 μl) contained 5 μl of SYBR Green Master Mix (Applied Biosystems), 1.5 μl of cDNA, 0.5 μl of forward and reverse primers (10 μM), and 2.5 μl of water. The thermal cycle condition for all reactions included 95°C denature for 10 min, 40 cycles at 95°C for 15 s and 60°C for 1 min, 95°C for 15 s, 60°C for 1 min, and 95°C for 15 s. Several PCR products mixed with DNA gel loading dye (×6; Thermo Fisher Scientific) from each gene were electrophoresed on a 3% agarose gel in Tris-acetate-EDTA buffer and visualized by 0.01% ethidium bromide, and a melting curve of each gene was checked to confirm the specificity of each PCR product. Glyceraldehyde 3-phosphate dehydrogenase (GAPDH) and beta actin were used as housekeeping genes (reference genes). The target mRNA abundance was normalized with geometric means of housekeeping genes (Vandesompele et al., 2002). Relative mRNA abundance was determined by using the 2^–∆∆Ct^ method (Livak and Schmittgen, 2001). The negative control, containing no cDNA, was included in each run, and each sample was run in duplicate.

**TABLE 2:** Primers used in the study.^1^.

Genes	Sequence, 5–3′	Amplicon
GAPDH	F: GCT AAG GCT GTG GGG AAA GT	161
	R: TCA GCA GCA GCC TTC ACT AC	
Beta actin	F: CAA CAC AGT GCT GTC TGG TGG TA	205
	R: ATC GTA CTC CTG CTT GCT GAT CC	
B0AT1	F: GGG TTT TGT GTT GGC TTA GGA A	60
	R: TCC ATG GCT CTG GCA GAG AT	
EAAT3	F: TGC TGC TTT GGA TTC CAG TGT	79
	R: AGC AAT GAC TGT AGT GCA GAA GTA ATA TAT G	
SGLT1	F: GCC ATG GCC AGG GCT TA	71
	R: CAA TAA CCT GAT CTG TGC ACC AGT A	
PEPT1	F: CCC CTG AGG AGG ATC CTT	66
	R: CAA AAG AGC AGC AAC GA	
MUC2	F: ATG CGA TGT TAA CAC AGG ACT C	110
	R: GTG GAG CAC AGC AGA CTT TG	
JAM2	F: AGC CTC AAA TGG GAT TGG ATT	59
	R: CAT CAA CTT GCA TTC GCT TCA	
ZO2	F: ATC CAA GAA GGC ACC TCA GC	100
	R: CAT CCT CCC GAA CAA TGC	
IL1β	F: TGC CTG CAG AAG AAG CCT CG	204
	R: GAC GGG CTC AAA AAC CTC CT	
IL2	F: TTG GCT GTA TTT CGG TAG CA	59
	R: GTG CAC TCC TGG GTC TCA GT	
IL6	F: ATA AAT CCC GAT GAA GTG G	146
	R: CTC ACG GTC TTC TCC ATA AA	
NFκB	F: GAA GGA ATC GTA CCG GGA ACA	131
	R: CTC AGA GGG CCT TGT GAC AGT AA	
ACACA	F: ACT​GAA​TTG​GTG​CTG​GAT​GA	103
	R: GGG​TCT​TGA​GGG​TCA​TTT​TC	
ACOX1	F: ATG​GAA​TTG​CAG​ACC​CAG​A	107
	R: GAG​AAG​GGT​AGG​GAG​GAA​CA	
APOB	F: CAA​ATG​CCA​TGT​CCA​AAC​A	110
	R: TCC​TCT​CTT​GAG​ATT​GAG​GAC​A	
CD36	F: CTG​GGA​AGG​TTA​CTG​CGA​TT	109
	R: GGA​TCT​GCA​AAT​GTC​AGA​GG	
CPT1A	F: CAG​ATG​TTA​TGA​CAG​GTG​GTT​TG	119
	R: CCC​ACA​GGT​GTC​CAA​CAA​TA	
FABP2	F: CTC​TTG​GAA​CCT​GGA​AGG​AA	125
	R: CTC​CTT​CGT​ACA​CGT​AGG​TCT​G	
FABP4	F: CAT​AAC​CCT​AGA​CAA​TGG​CAC​A	101
	R: ATTCCACCAGCAGGTTCC	
FASN	F: GGC​AAC​TAT​CCT​TCC​CAA​AA	118
	R: GAG​GGA​GAT​CTT​CCC​ACT​CA	
GCG	F: AAG​AAA​TGG​CCA​ACA​AGG​AC	107
	R: GCC​TTC​AGC​ATG​TCT​CTC​AA	
PPARγ	F: GAT​GAC​AGT​GAC​CTG​GCA​AT	116
	R: TCC​AAA​GCT​TGC​AAC​AGA​TT	

1 GAPDH, glyceraldehyde 3-phosphate dehydrogenase; B0AT1, sodium-dependent neutral amino acid transporter 1; EAAT3, excitatory amino acid transporter 3; SGLT1, sodium glucose transporter 1; PepT1, peptide transporter 1; MUC2, mucin 2; JAM2, Junctional adhesion molecule 2; Zonula occludens 2; zonula occludens 2; IL, interleukin; NFκB, nuclear factor kappa-light-chain-enhancer of activated B cells; ACACA, acetyl-CoA carboxylase alpha; ACOX1, acyl-CoA oxidase 1; APOB, apolipoportine B; CD36, clusters of differentiation 36; CPT1A, carnitine palmitoyltransferase 1A; FABP, fatty acid binding protein; FASN, fatty acid synthase; GCG, glucacon; PPARγ, peroxisome proliferator-activated receptor gamma.

### Total antioxidant capacity, glutathione (GSH), oxidized GSH (GSSG), and superoxide dismutase in the liver

Approximately 100 mg of the liver samples were homogenized with 1 ml of designated solution for each analysis including total antioxidant capacity (TAC), glutathione (GSH) and oxidized GSH (GSSG), and superoxide dismutase (SOD) using a beads beater (Biospec Products, Bartlesville, OK, United States). The TAC of the liver tissues were determined using colorimetric microplate assay kits for Total antioxidant capacity (TA02, Oxford Biomedical Research, Oxford, MI, United States) according to Choi et al. (2020b). The GSH and GSSG in the liver were analyzed by using Caymans GSH assay kits (Cayman Chemical, Ann Arbor, MI, United States) with a 20-time sample dilution. The activities of SOD in the liver were analyzed using Caymans SOD assay kit (Cayman Chemical) with a 400-time sample dilution. The supernatants of homogenized samples after the centrifugation at 4°C and 12,000 × g for 15 min were collected to analyze their protein content using Pierce BCA protein assay kits (Thermo Fisher Scientific) with a 20-time sample dilution. The TAC, GSH and GSSG concentrations, and SOD activities were expressed as values per mg protein.

### Body composition analyses by using dual-energy X-ray absorptiometry

On D 21, three birds per pen were euthanized by cervical dislocation. According to Wang et al. (2021b), dual-energy X-ray absorptiometry (DEXA, GE Healthcare, Madison, WI, United States) was used to measure bone mineral density (g/cm2), bone mineral content (g), tissue weight (g), fat weight (g), lean weight (g), body fat percentage (%), and lean:fat (g/g).

### Cecal volatile fatty acid analysis

Concentrations of volatile fatty acids (VFA) in cecal digesta were determined according to the method of Choi et al. (2021). The samples were snap-frozen in liquid nitrogen and stored at −80°C for further analyses. Once thawed, 0.5 g samples were diluted with 3 ml distilled water. The samples were vigorously homogenized for 1 min and subsequently frozen at −20°C. After the samples were thawed, the samples were centrifuged at 10,000 × g for 10 min, and 850 μl of the supernatants were collected, mixed with 170 μl of the fresh 25% (wt/vol) meta-phosphoric acid solution, and immediately frozen at −20°C overnight. The samples were centrifuged at 10,000 × g for 10 min, and 800 μl of the supernatant was collected and mixed with 1,600 μl ethyl acetate. The samples were vigorously homogenized for 10 s and allowed to settle for 5 min. The top layer was transferred to a screw-thread vial and analyzed in a gas chromatograph (Shimadzu GC-2010 plus; Shimadzu Corporation, Kyoto, Japan) equipped with an autoinjector (AOC-20i; Shimadzu Corporation, Kyoto, Japan). A capillary column (Zebron ZB-FFAP; 30 m × 0.32 mm × 0.25 μm; Phenomenex Inc., Torrance, CA, United States) was utilized for the separation of the VFA. The sample injection volume was set at 1 ml, and helium was used as the carrier gas. The column temperature was initially set at 110°C and gradually increased to 200°C over the course of 6 min. The flame ionization detector was set at 350°C.

### DNA extraction and microbiome analysis in the cecal content

DNA was extracted from the contents of mid-ceca by using QIAamp^®^ DNA stool mini kits (Qiagen GmbH, Hilden, Germany) according to manufacturer procedure. Quality and quantity of extracted DNA was checked using a NanoDrop 2000spectrophotometer (Thermo Fisher Scientific). The 16s rRNA gene sequencing was conducted by LC sciences, LLC (Houston, TX, United States). The V3 and V4 regions were amplified using 338F (5-CCTACGGGNGGCWGCAG-3)/806R (5-GACTACHVGGGTATCTAATCC-3) primers by PCR procedures as follows. The 5′ ends of the primers were tagged with specific barcodes per sample and sequencing universal primers. The PCR amplification was conducted in a total volume of 25 μl reaction mixture containing 25 ng of template DNA, 12.5 μl PCR Premix, 2.5 μl of each primer, and PCR-grade water to adjust the volume. The PCR conditions to amplify the prokaryotic 16S fragments consisted of an initial denaturation at 98°C for 30 s; 32 cycles of denaturation at 98°C for 10 s, annealing at 54°C for 30 s, and extension at 72°C for 45 s; and then final extension at 72°C for 10 min. The PCR products were checked with 2% agarose gel electrophoresis. The PCR products were purified using AMPure XT beads (Beckman Coulter Genomics, Danvers, MA, United States) and quantified by Qubit (Invitrogen, Carlsbad, CA, United States). The amplicon pools were prepared for sequencing, and the size and quantity of the amplicon library were assessed on Agilent 2100 Bioanalyzer (Agilent, Palo Alto, CA, United States) and with the library quantification kit for Illumina (Kapa Biosciences, Woburn, MA, United States), respectively. The libraries were sequenced on NovaSeq PE250 platform. For 16s rRNA analyses, QIIME2 (version 2021.11) was used (Bolyen et al., 2019). By using the QIIME2 demux emp-paired function and QIIME2 plugin DADA2, sequences were demultiplexed and denoised, respectively. The lowest frequency was 14,500, and the highest frequency was 32,278 after trimming the sequences of all samples. GreenGenes database (version 13.8) at the 99% operational taxonomic units (OTUs) for the region (515F/806R) was used for taxonomical classification. Phylum- and family-level composition were presented. Alpha diversity and beta diversity were analyzed by QIIME2’s built in functions. Heatmap of the top 30 most abundant features was generated by QIIME2’s built in functions.

### Statistical analyses

SAS (version 9.4; SAS Inst. Inc., Cary, NC, United States) and GraphPad Prism (Version 9.1.0; GraphPad Software, San Diego, CA, United States) were utilized for statistical analyses and graph construction. Treatment groups were compared using one-way ANOVA in a completely randomized design followed by the Tukey’s comparison test. Orthogonal polynomial contrasts were used to evaluate the significance of linear or quadratic effects of supplemental TA in broilers. To calculate the optimal dosage of TA in broilers, the segmented linear regression analysis was conducted, and inflection point (*X0*) with *p* values for slope 1 (<*X0*) and slope 2 (>*X0*) were presented. Significance level was set at *p* < 0.05, and tendencies were also presented at 0.05 < *p* ≤ 0.10 (Choi et al., 2021). Correlation between the cecal microbial composition with VFA and growth performance, fat accumulation, bone health, fat metabolism mRNA expression, immune system, gut barrier integrity, brush border digestive enzymes, antioxidant capacity, AID, nutrient transporters, and VFA parameters were analyzed, parameters with significant differences (*p* < 0.05) were presented.

## Results

### Growth performance

**FIGURE 1:**
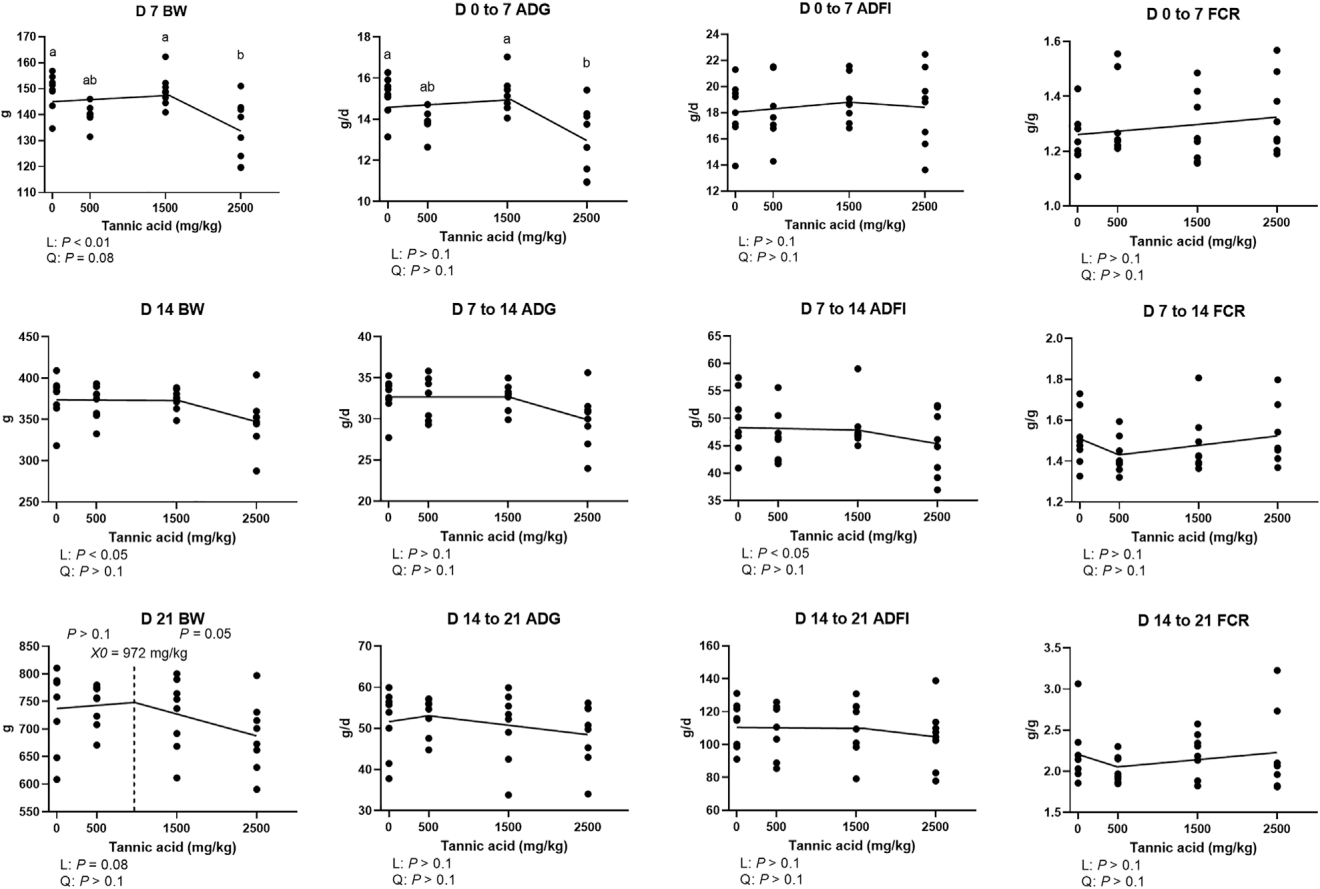
Growth performance parameters (BW, ADG, ADFI, and FCR) of broiler chickens fed different concentrations (0, 0.5, 1.5, and 2.5 g/kg) of tannic acid on D 7, 14, and 21. The treatment groups were compared using one-way ANOVA followed by the Tukey’s multiple comparison. Different superscripts differ at *p* < 0.05. Orthogonal polynomial contrasts analysis was conducted to see linear pattern (L) and quadratic pattern (Q) among the treatments. To calculate the optimal dosage of tannic acid in broilers, segmented linear regression was conducted, and *X0* (inflection point) with *p* values for slope 1 (lower than *X0*) and slope 2 (higher than *X0*) were presented.

The results of growth performance in broilers fed different concentrations of TA are presented in Figure 1. On D 7, the TA2.5 group had lower BW compared to the TA0 group (*p* < 0.05), and supplemental TA linearly (*p* < 0.05) and quadratically (tendency; *p* = 0.08) modulated BW. On D 14, BW and ADFI were linearly reduced by supplemental TA (*p* < 0.05). On D 21, supplemental TA tended to reduce BW (*p* = 0.08), and at levels greater than 972 mg/kg supplemental TA tended to reduce BW in broilers (*p* = 0.08).

### Apparent ileal digestibility of dry matter, organic matter, ash, crude protein, and ether extract

**FIGURE 2:**
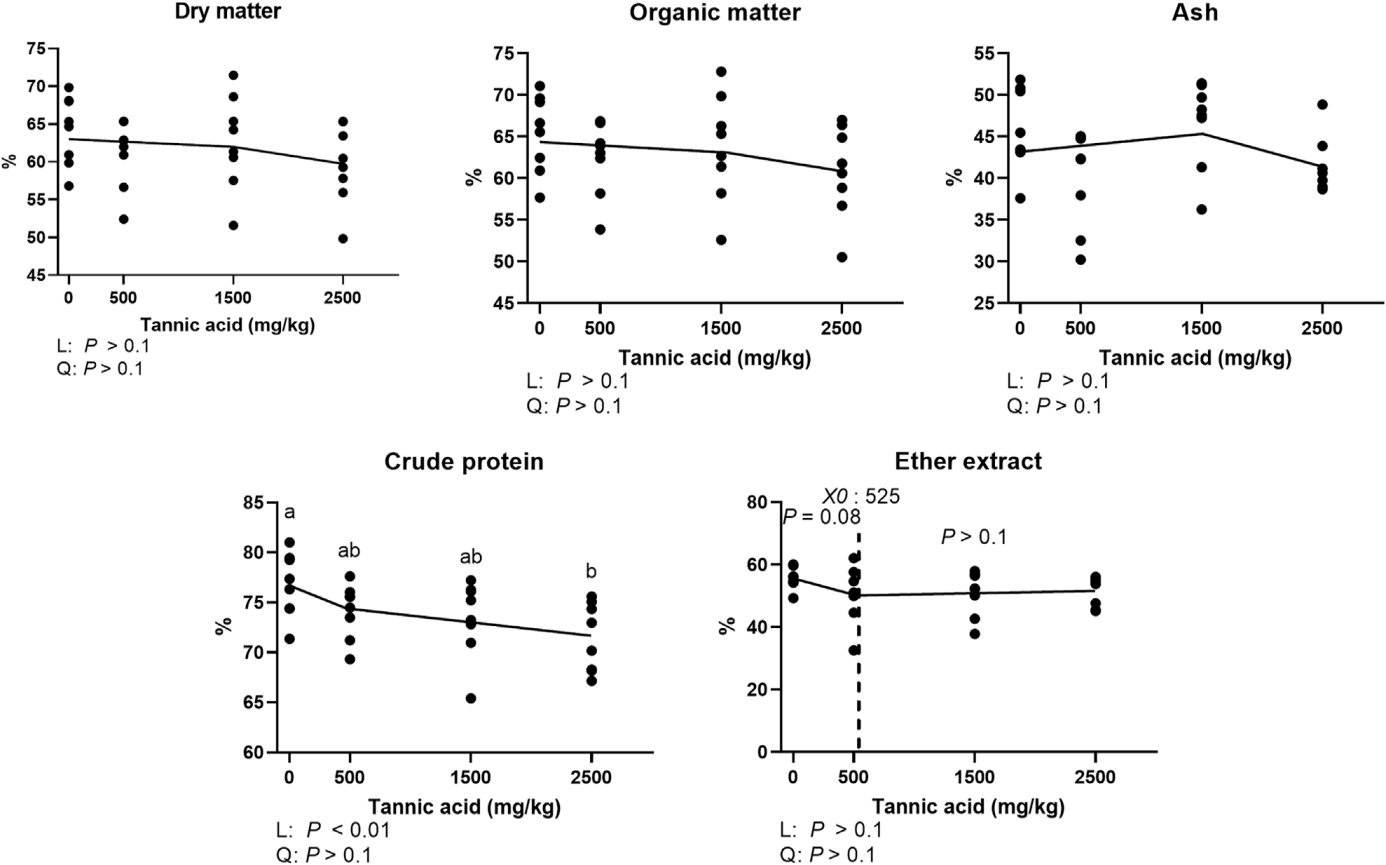
Apparent ileal digestibility of dry matter, organic matter, ash, crude protein, ether extract in broiler chickens fed different concentrations (0, 0.5, 1.5, and 2.5 g/kg) of tannic acid on D 21. The treatment groups were compared using one-way ANOVA followed by the Tukey’s multiple comparison. Different superscripts differ at *p* < 0.05. Orthogonal polynomial contrasts analysis was conducted to see linear pattern (L) and quadratic pattern (Q) among the treatments. To calculate the optimal dosage of tannic acid in broilers, segmented linear regression was conducted, and *X0* (inflection point) with *p* values for slope 1 (lower than *X0*) and slope 2 (higher than *X0*) were presented.

Tthe results of AID of DM, OM, ash, CP, and EE in broilers fed different concentrations of TA are shown in Figure 2. The AID of DM, OM, and ash was not affected by supplemental TA (Figure 2; *p* > 0.1). However, AID of CP was significantly reduced by supplemental TA (*p* < 0.01). The AID of EE tended to be reduced by TA at levels less than 525 mg/kg (*p* = 0.08).

### Jejunal and cecal morphology and goblet cell density

**FIGURE 3:**
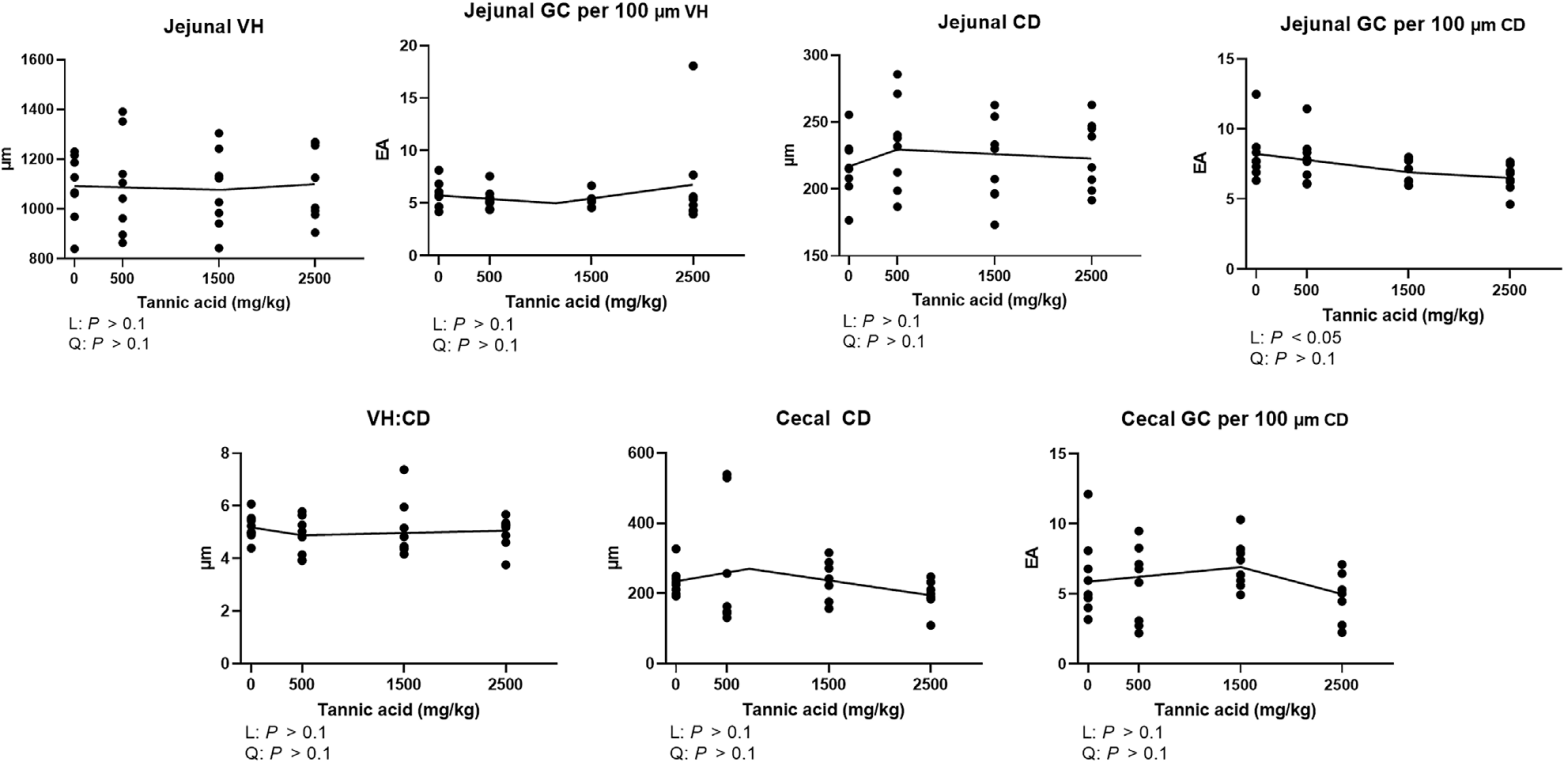
Jejunal morphology [villus height (VH), crypts depth (CD), and VH:CD] and goblet cell (GC) counting per 100 μm VH and CD and cecal CD and goblet cell counting per 100 μm CD in broiler chickens fed different concentrations (0, 0.5, 1.5, and 2.5 g/kg) of tannic acid on D 21. The treatment groups were compared using one-way ANOVA followed by the Tukey’s multiple comparison. Different superscripts differ at *p* < 0.05. Orthogonal polynomial contrasts analysis was conducted to see linear pattern (L) and quadratic pattern (Q) among the treatments. To calculate the optimal dosage of tannic acid in broilers, segmented linear regression was conducted, and *X0* (inflection point) with *p* values for slope 1 (lower than *X0*) and slope 2 (higher than *X0*) were presented.

Jejunal and cecal morphology and goblet cell density in the broilers fed different concentrations of TA are presented in Figure 3. No differences were observed in the jejunal VH and CD and cecal CD (*p* > 0.1). However, jejunal goblet cell number per 100 μm CD was significantly reduced by supplemental TA (*p* < 0.05).

### Activities of jejunal brush border digestive enzymes and serum alkaline phosphatase

**FIGURE 4:**
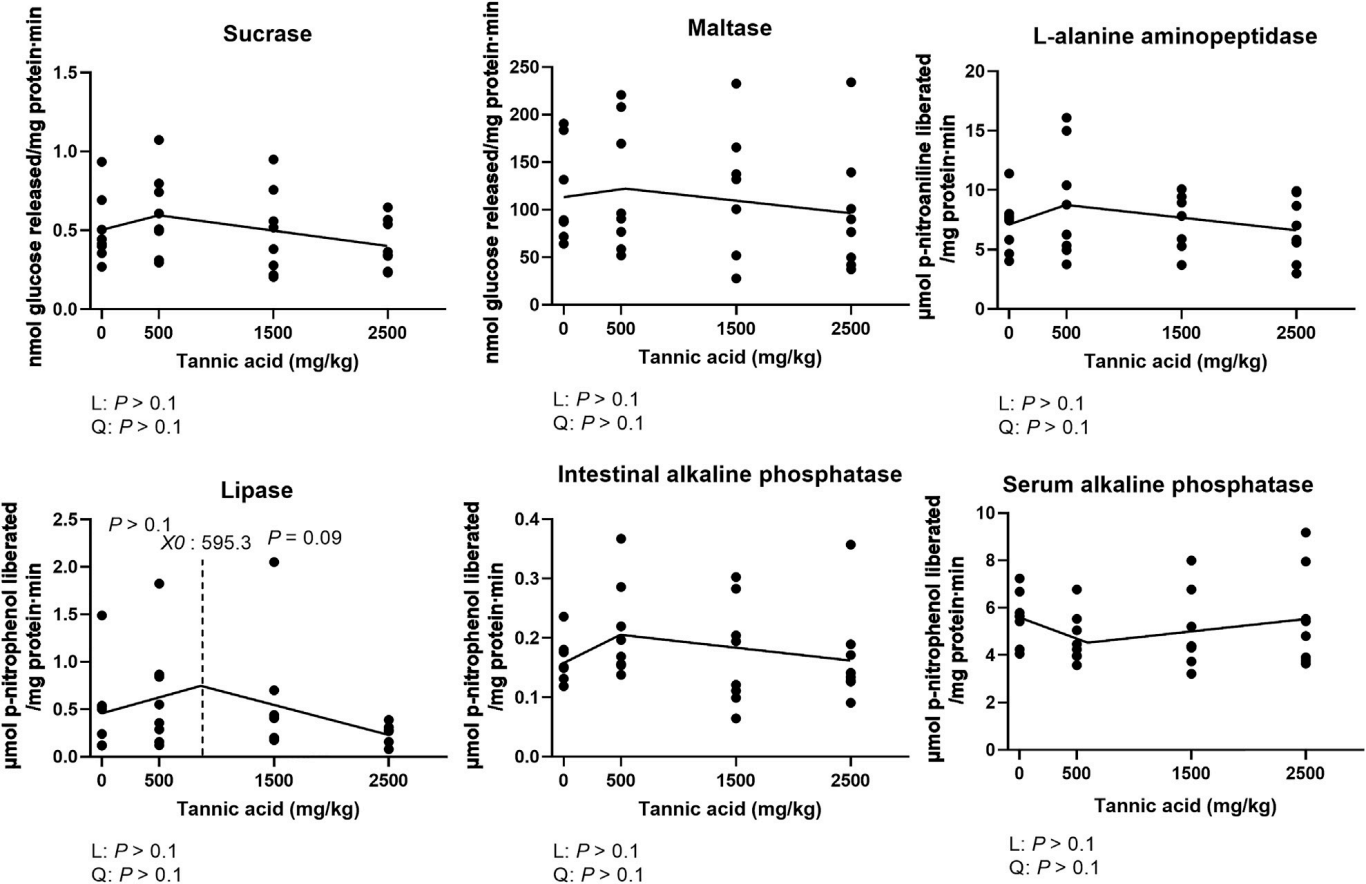
Activities of sucrase, maltase, L-alanine aminopeptidase, intestinal alkaline phosphatase, serum alkaline phosphatase lipase in the jejunum in broiler chickens fed different concentrations (0, 0.5, 1.5, and 2.5 g/kg) of tannic acid on D 21. The treatment groups were compared using one-way ANOVA followed by the Tukey’s multiple comparison. Different superscripts differ at *p* < 0.05. Orthogonal polynomial contrasts analysis was conducted to see linear pattern (L) and quadratic pattern (Q) among the treatments. To calculate the optimal dosage of tannic acid in broilers, segmented linear regression was conducted, and *X0* (inflection point) with *p* values for slope 1 (lower than *X0*) and slope 2 (higher than *X0*) were presented.

Activities of jejunal maltase, sucrase, APN, and IAP were not altered by supplemental TA in broilers (Figure 4; *p* > 0.1). However, the activities of lipase showed a tendency to be reduced by TA at levels greater than 595.3 mg/kg (*p* = 0.09). Activities of alkaline phosphatase in the serum were not affected by supplemental TA (Figure 4; *p* > 0.1).

### Relative mRNA expression of genes related to nutrient transportation, gut barrier integrity, and inflammation in the jejunum

**FIGURE 5:**
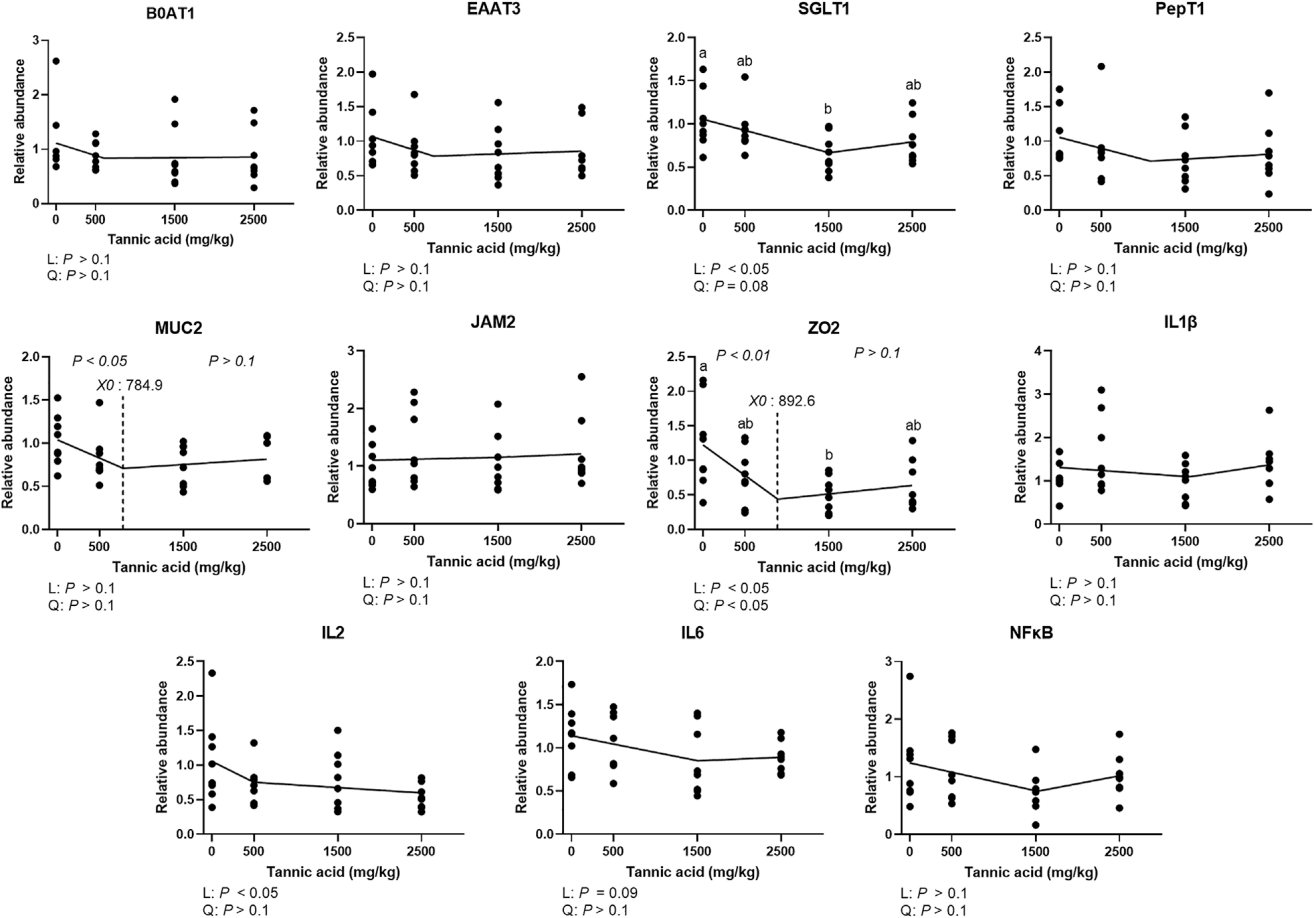
Relative mRNA expression of genes associated with nutrient transportation, gut barrier integrity, and inflammation in the jejunum in broiler chickens fed different concentrations (0, 0.5, 1.5, and 2.5 g/kg) of tannic acid on D 21. The treatment groups were compared using one-way ANOVA followed by the Tukey’s multiple comparison. Different superscripts differ at *p* < 0.05. Orthogonal polynomial contrasts analysis was conducted to see linear pattern (L) and quadratic pattern (Q) among the treatments. To calculate the optimal dosage of tannic acid in broilers, segmented linear regression was conducted, and *X0* (inflection point) with *p* values for slope 1 (lower than *X0*) and slope 2 (higher than *X0*) were presented. B0AT1, sodium-dependent neutral amino acid transporter 1; EAAT3, excitatory amino acid transporter 3; SGLT1, sodium glucose transporter 1; PepT1, peptide transporter 1; MUC2, mucin 2; JAM2, Junctional adhesion molecule 2; Zonula occludens 2; zonula occludens 2; IL, interleukin; NFκB, nuclear factor kappa-light-chain-enhancer of activated B cells.

The relative mRNA expression of nutrient transporters, tight junction proteins, and cytokines in broilers fed different concentrations of TA is presented in Figure 5. No differences were observed in the mRNA expression of sodium-dependent neutral amino acid transporter 1 (B0AT1), excitatory amino acid transporter 3 (EAAT3), peptide transporter 1 (PepT1), junctional adhesion molecule 2 (JAM2), interleukins 1β (IL1β), and nuclear factor kappa-light-chain-enhancer of activated B cells (NFκB) (*p* > 0.1). Supplemental TA linearly (*p* < 0.05) and quadratically (tendency; *p* = 0.08) reduced mRNA expression of sodium glucose transporter 1 (SGLT1), and the TA1.5 group had significantly lower mRNA expression of SGLT1 compared to the TA0 group. The mRNA expression of mucin 2 (MUC2) was linearly decreased by TA at levels less than 784.9 mg/kg (*p* < 0.05). The mRNA expression of zonula occludens 2 (ZO2) was linearly and quadratically modulated (*p* < 0.05) and was linearly reduced by supplemental TA at levels less than 892.6 mg/kg (*p* < 0.01), and the TA1.5 group had significantly lower mRNA of ZO2 compared to the TA0 group. Supplemental TA linearly decreased mRNA expression of IL2 (*p* < 0.05) and IL6 (tendency; *p* = 0.09).

### 3.6 Total antioxidant capacity, activities of superoxide dismutase, glutathione and oxidized glutathione concentrations in the liver

**FIGURE 6:**
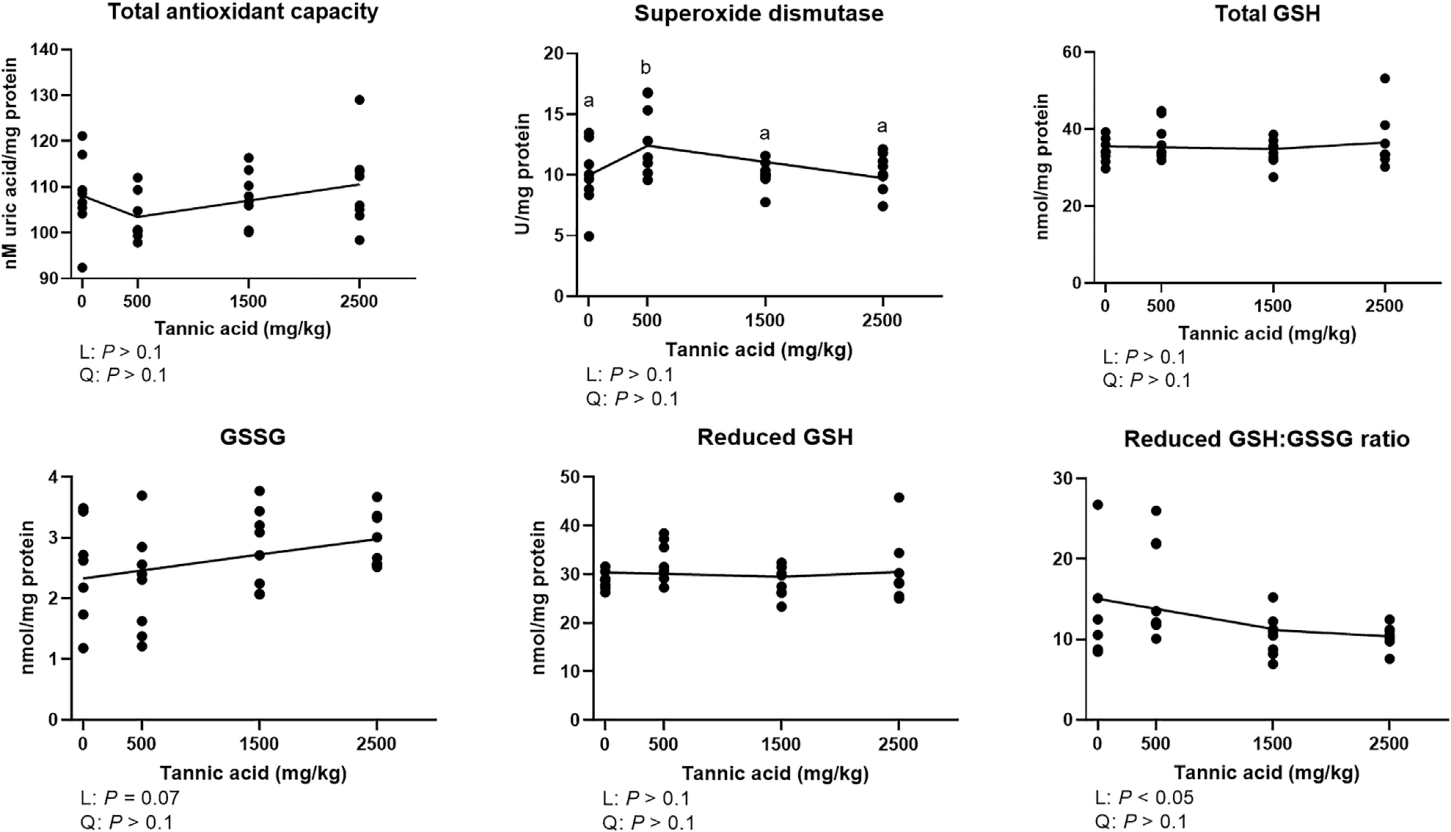
Total antioxidant capacity, glutathione (GSH) and oxidized glutathione (GSSG) concentrations, and activities of superoxide dismutase (SOD) in broiler chickens fed different concentrations (0, 0.5, 1.5, and 2.5 g/kg) of tannic acid on D 21. The treatment groups were compared using one-way ANOVA followed by the Tukey’s multiple comparison. Different superscripts differ at *p* < 0.05. Orthogonal polynomial contrasts analysis was conducted to see linear pattern (L) and quadratic pattern (Q) among the treatments. To calculate the optimal dosage of tannic acid in broilers, segmented linear regression was conducted, and *X0* (inflection point) with *p* values for slope 1 (lower than *X0*) and slope 2 (higher than *X0*) were presented. Reduced GSH = Total GSH – 2 × GSSG.

Total antioxidant capacity was not modulated by supplemental TA (Figure 6; *p* > 0.1). The TA0.5 group had the highest activities of SOD among the treatment groups (*p* < 0.05). Although there were no differences in total GSH, and reduced GSH, supplemental TA showed a tendency to linearly increase GSSG in broilers (*p* = 0.07) and linearly decreased reduced GSH:GSSG (*p* < 0.05).

### Bone mineral density and body composition analyses

**FIGURE 7:**
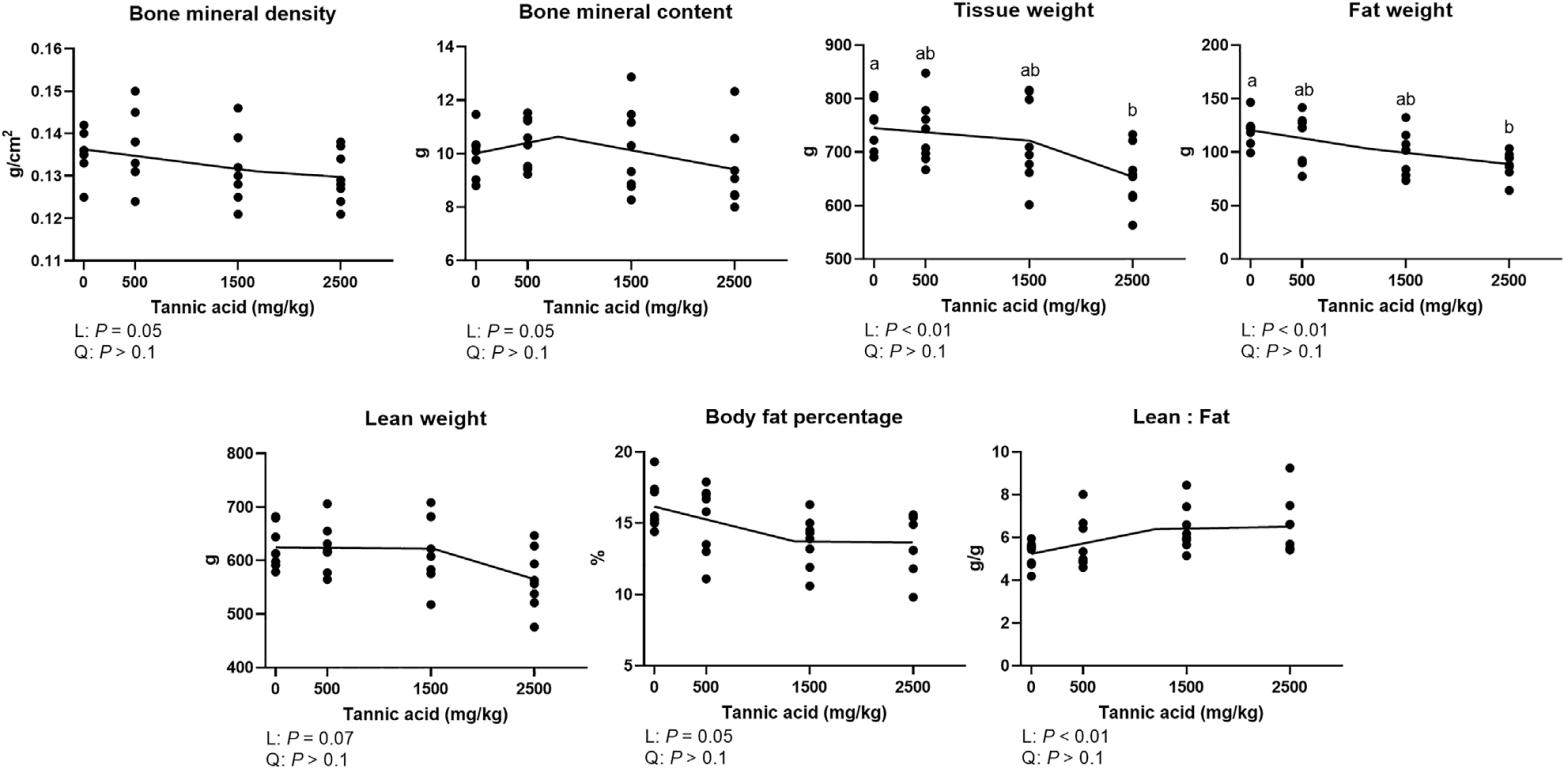
Bone mineral density (g/cm^2^), bone mineral content (g), tissue weight (g), fat weight (g), lean weight (g), body fat percentage (%), and lean:fat (g/g) in broiler chickens fed different concentrations (0, 0.5, 1.5, and 2.5 g/kg) of tannic acid on D 21. The treatment groups were compared using one-way ANOVA followed by the Tukey’s multiple comparison. Different superscripts differ at *p* < 0.05. Orthogonal polynomial contrasts analysis was conducted to see linear pattern (L) and quadratic pattern (Q) among the treatments. To calculate the optimal dosage of tannic acid in broilers, segmented linear regression was conducted, and *X0* (inflection point) with *p* values for slope 1 (lower than *X0*) and slope 2 (higher than *X0*) were presented.

The bone mineral density and body composition indices in broilers fed different concentrations of TA are presented in Figure 7. Bone mineral density (tendency; *p* = 0.05) and contents (tendency; *p* = 0.05) were linearly reduced as supplemental TA levels increased. Tissue weight was linearly reduced (*p* < 0.05) by supplemental TA, and TA2.5 had the lower tissue weight compared to the TA0 group (*p* < 0.05). Fat weight was linearly reduced (*p* < 0.01), and the TA2.5 group had lower fat weight compared to the TA0 group (*p* < 0.05). Body fat percentage (*p* = 0.05) and lean weight (*p* = 0.07) tended to be reduced by supplemental TA. The lean to fat ratio was linearly increased as supplemental TA levels increased (*p* < 0.01).

### Relative mRNA expression of genes related to fat metabolism in the liver

**FIGURE 8:**
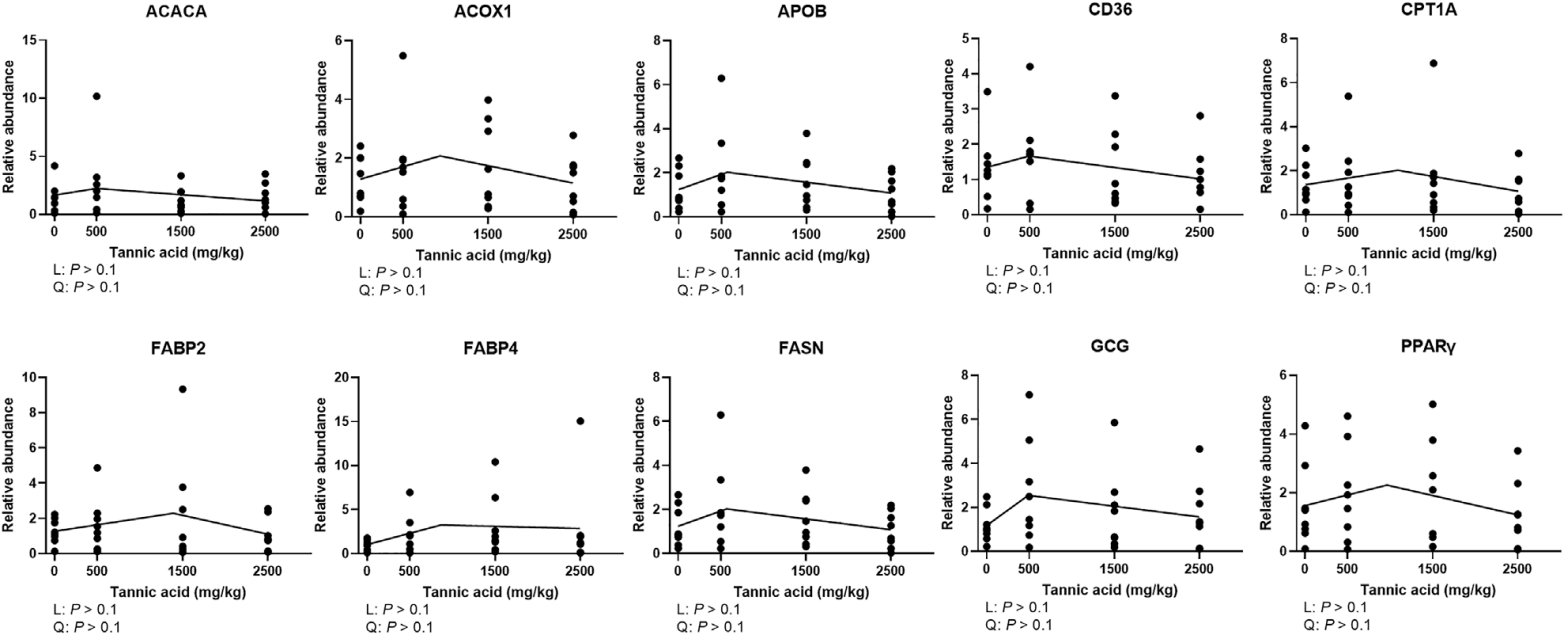
Relative mRNA expression of genes related to fat metabolism in the liver of broiler chickens fed different concentrations (0, 0.5, 1.5, and 2.5 g/kg) of tannic acid on D 21. The treatment groups were compared using one-way ANOVA followed by the Tukey’s multiple comparison. Different superscripts differ at *p* < 0.05. Orthogonal polynomial contrasts analysis was conducted to see linear pattern (L) and quadratic pattern (Q) among the treatments. To calculate the optimal dosage of tannic acid in broilers, segmented linear regression was conducted, and *X0* (inflection point) with *p* values for slope 1 (lower than *X0*) and slope 2 (higher than *X0*) were presented. ACACA, acetyl-CoA carboxylase alpha; ACOX1, acyl-CoA oxidase 1; APOB, apolipoportine B; CD36, clusters of differentiation 36; CPT1A, carnitine palmitoyltransferase 1A; FABP, fatty acid binding protein; FASN, fatty acid synthase; GCG, glucacon; PPARγ, peroxisome proliferator-activated receptor gamma.

Supplemental TA did not modulate mRNA expression of genes related to fat metabolism in the liver of broilers (Figure 8; *p* > 0.1).

### Cecal volatile fatty acid composition

**FIGURE 9:**
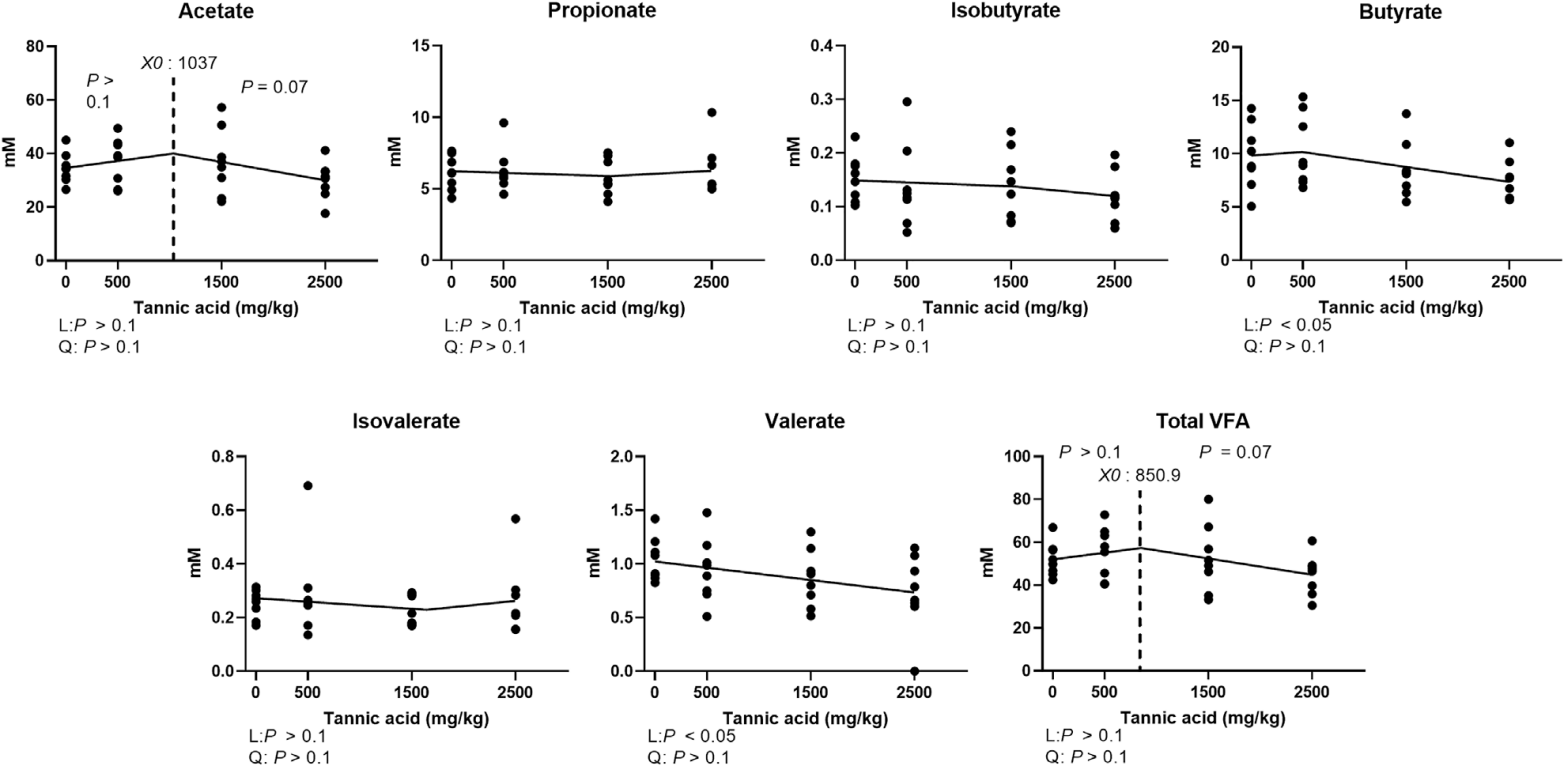
Cecal volatile fatty acid concentration (mM) in broiler chickens fed different concentrations (0, 0.5, 1.5, 2.5 g/kg) of tannic acid on D 21. The treatment groups were compared using one-way ANOVA followed by the Tukey’s multiple comparison. Different superscripts differ at *p* < 0.05. Orthogonal polynomial contrasts analysis was conducted to see linear pattern (L) and quadratic pattern (Q) among the treatments. To calculate the optimal dosage of tannic acid in broilers, segmented linear regression was conducted, and *X0* (inflection point) with *p* values for slope 1 (lower than *X0*) and slope 2 (higher than *X0*) were presented.

Modulated cecal VFA composition in broilers fed different concentrations of TA in Figure 9. The acetate concentration tended to be reduced at TA levels greater than 1,037 mg/kg were supplemented (*p* = 0.07). Supplemental TA linearly decreased the concentrations of butyrate and valerate (*p* < 0.05). Total VFA concentrations tended to be reduced by TA at levels greater than 850.9 mg/kg (*p* = 0.07).

### Alpha and beta diversity of the cecal microbial communities

**FIGURE 10:**
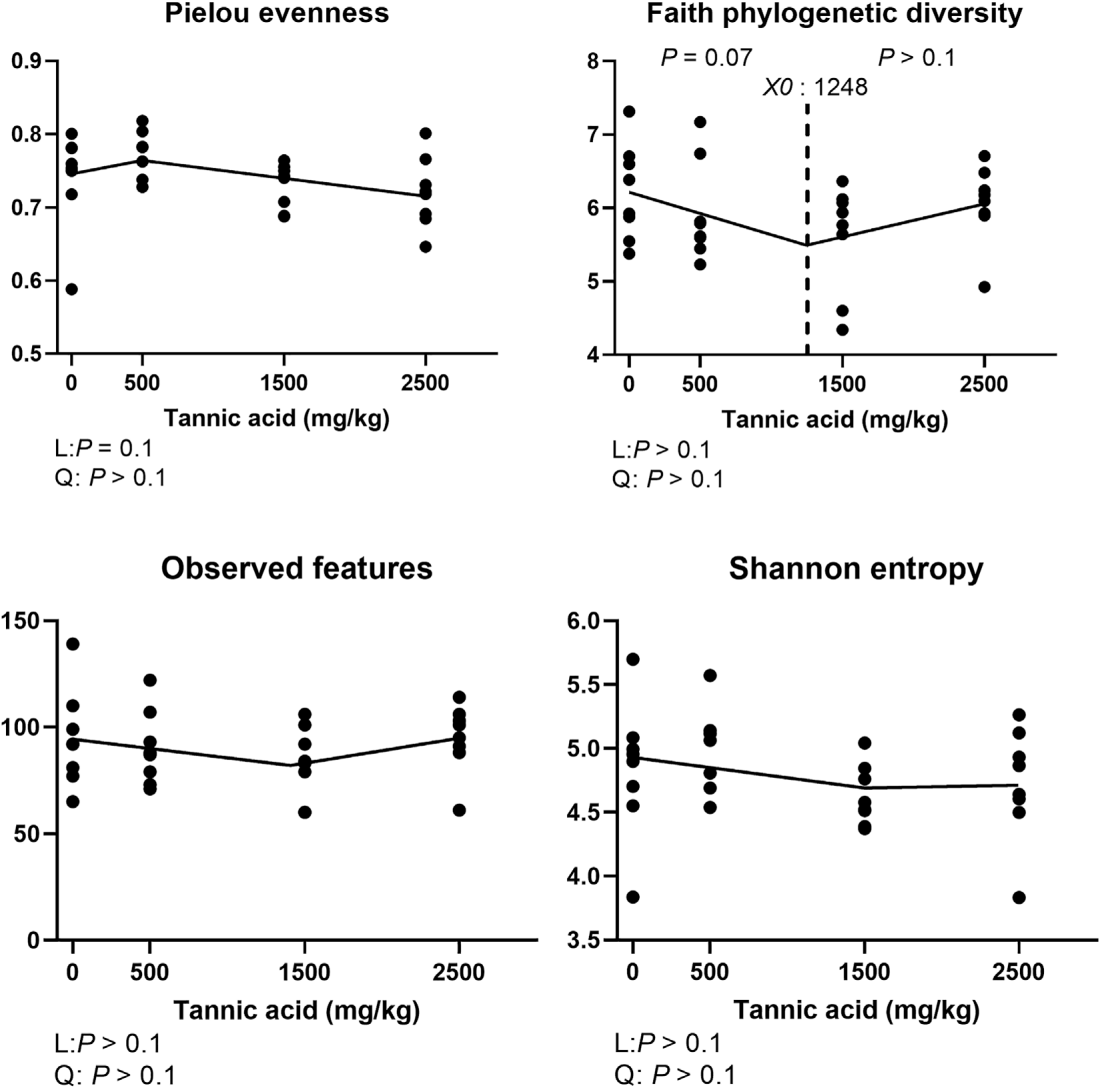
Alpha diversity indices of the cecal microbial communities in broiler chickens fed different concentrations (0, 0.5, 1.5, and 2.5 g/kg) of tannic acid on D 21. The treatment groups were compared using one-way ANOVA followed by the Tukey’s multiple comparison. Different superscripts differ at *p* < 0.05. Orthogonal polynomial contrasts analysis was conducted to see linear pattern (L) and quadratic pattern (Q) among the treatments. To calculate the optimal dosage of tannic acid in broilers, segmented linear regression was conducted, and *X0* (inflection point) with *p* values for slope 1 (lower than *X0*) and slope 2 (higher X0) were presented.

The alpha diversity indices including pielou evenness, faith phylogenetic diversity, observed features, and shannon entropy are presented in Figure 10. Supplemental TA tended to linearly decrease pielou evenness (*p* = 0.1). Faith phylogenetic diversity tended to be reduced by TA at levels greater than 1,248 mg/kg (*p* = 0.07). However, there were no differences in observed features and shannon entropy.

**FIGURE 11:**
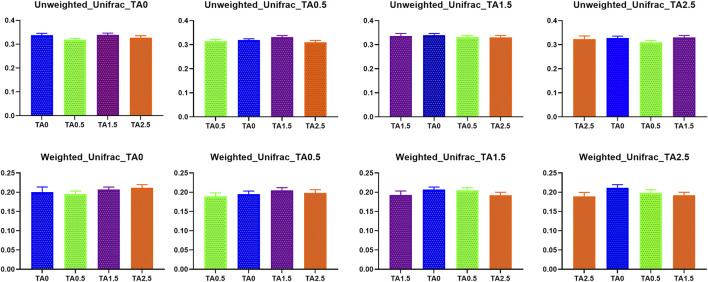
Beta diversity indices (Weighted and unweighted Unifrac) of the cecal microbial communities in the broilers of the tannic acid 0 (TA0): basal diet without tannic acid; 2) tannic acid 0.5 (TA0.5): basal diet with 0.5 g/kg tannic acid; 3) tannic acid 1.5 (TA1.5); and 4) tannic acid 2.5 (TA2.5) groups on D 21. The treatment groups were compared using one-way ANOVA followed by the Tukey’s multiple comparison. Different superscripts differ at *p* < 0.05.

**FIGURE 12:**
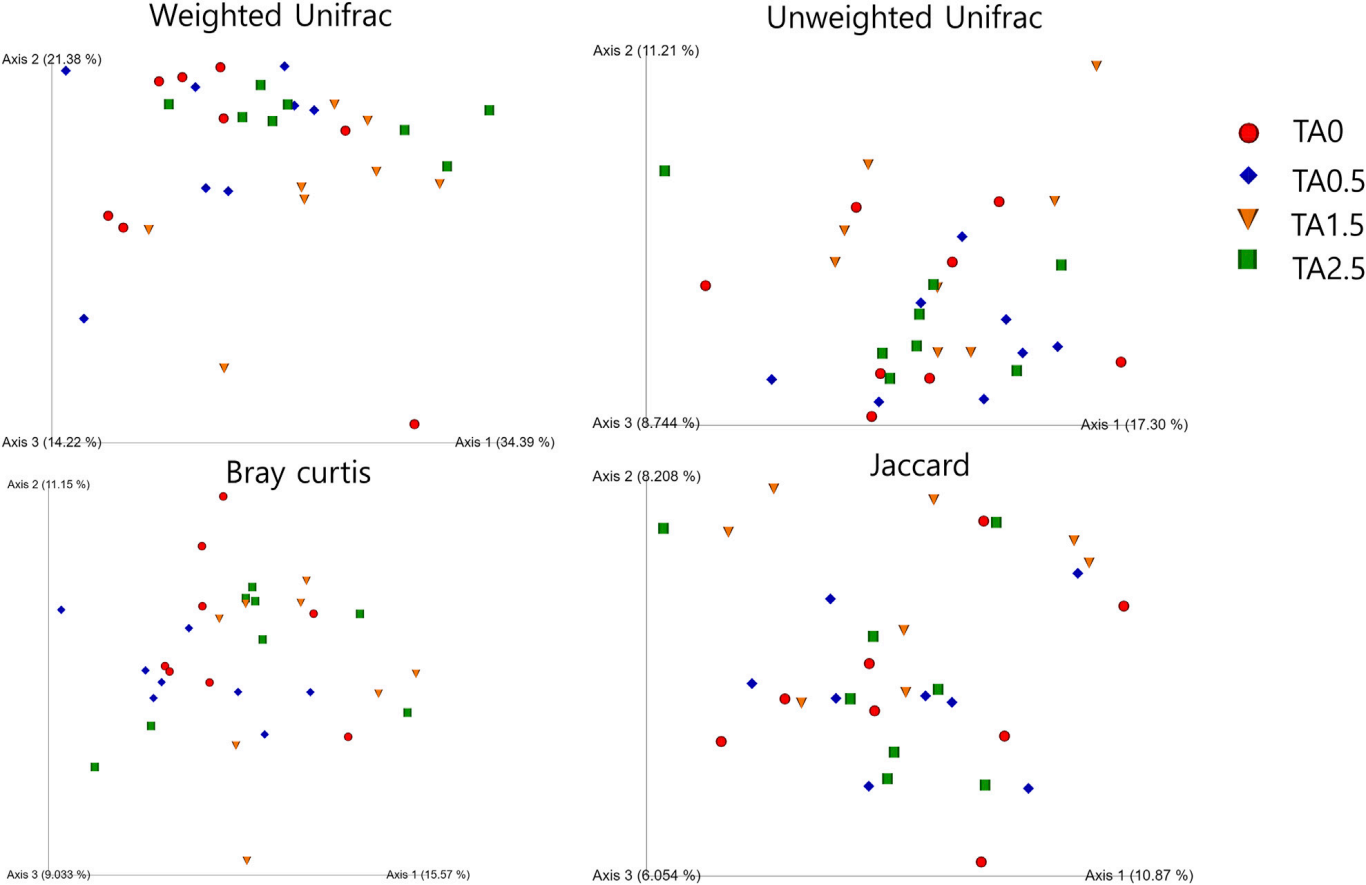
Visualization of beta diversity indices (weighted and unweighted Unifrac, bray curtis, and jaccard) in the broilers of the tannic acid 0 (TA0): basal diet without tannic acid; 2) tannic acid 0.5 (TA0.5): basal diet with 0.5 g/kg tannic acid; 3) tannic acid 1.5 (TA1.5); and 4) tannic acid 2.5 (TA2.5) groups on D 21. The treatment groups were compared using one-way ANOVA followed by the Tukey’s multiple comparison. Different superscripts differ at *p* < 0.05.

Beta diversity indices including weighted and unweighted unifrac are presented in Figure 11. No statistical differences were observed in those indices. Visualized beta diversity indices such as weighted emperor, unweighted emperor, bray curtis, and jaccard did not show any obvious differences (Figure 12).

### Phylum- and family-level composition of the cecal microbial communities

**FIGURE 13:**
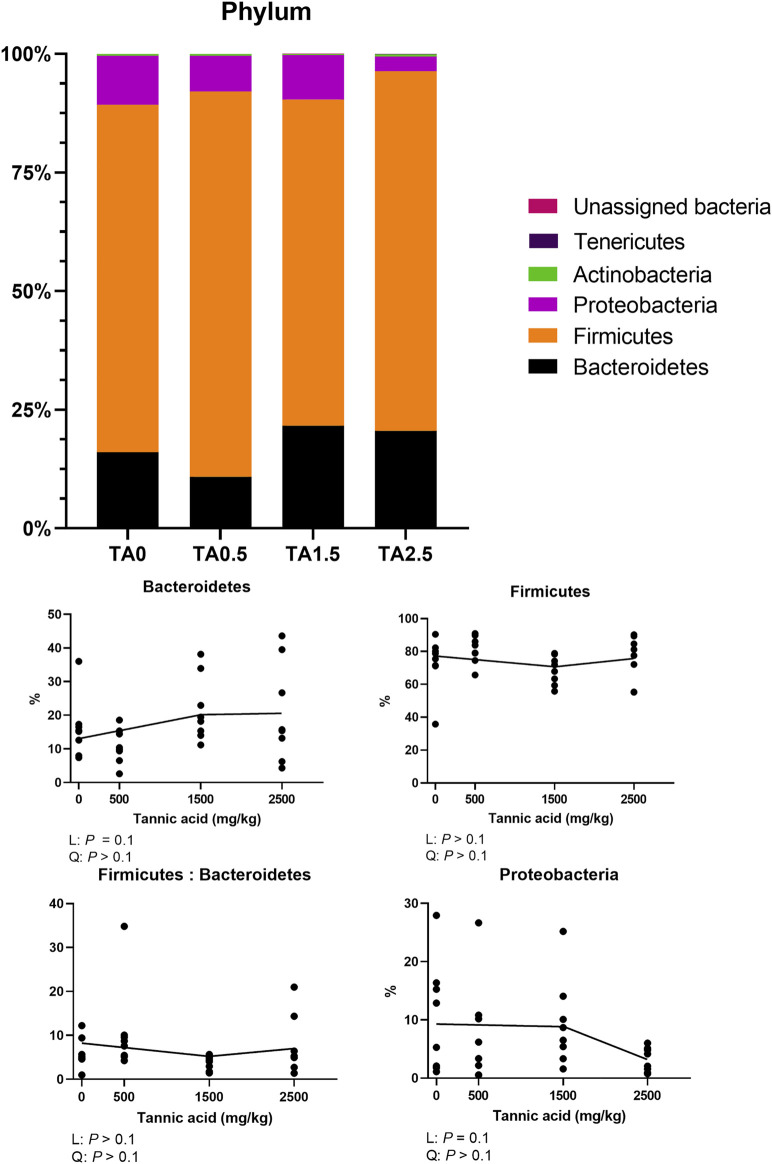
Phylum-level composition of the cecal microbial communities in broiler chickens fed different concentrations (0, 0.5, 1.5, and 2.5 g/kg) of tannic acid on D 21. Only phylum with significant differences and relatively high relative abundance were presented separately with statistical analyses. The treatment groups were compared using one-way ANOVA followed by the Tukey’s multiple comparison. Different superscripts differ at *p* < 0.05. Orthogonal polynomial contrasts analysis was conducted to see linear pattern (L) and quadratic pattern (Q) among the treatments. To calculate the optimal dosage of tannic acid in broilers, segmented linear regression was conducted, and *X0* (inflection point) with *p* values for slope 1 (lower than *X0*) and slope two higher X0) were presented.

**FIGURE 14:**
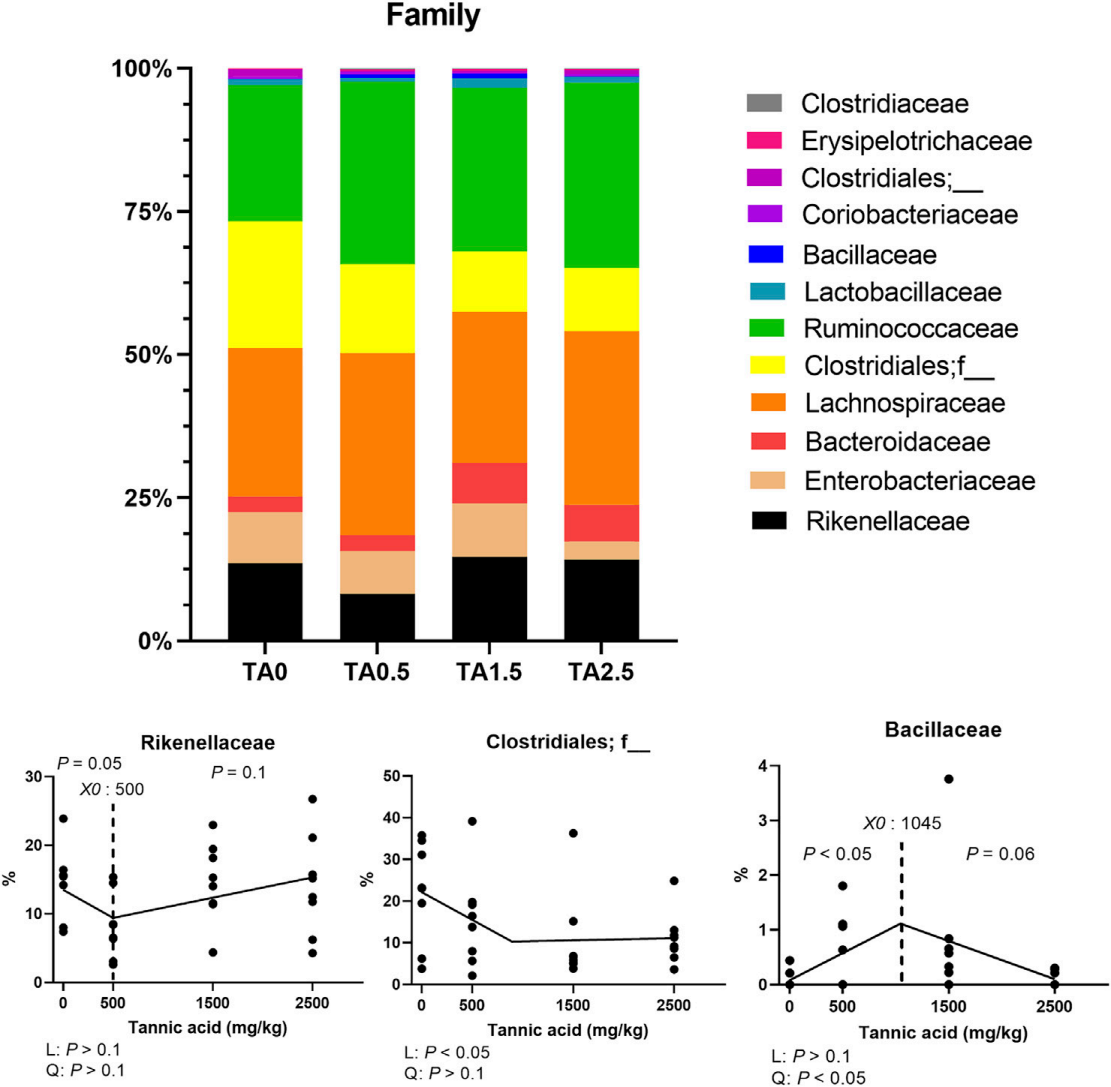
Family-level composition of the cecal microbial communities in broiler chickens fed different concentrations (0, 0.5, 1.5, and 2.5 g/kg) of tannic acid on D 21. Only families with significant differences and relatively high relative abundance were presented separately with statistical analyses. The treatment groups were compared using one-way ANOVA followed by the Tukey’s multiple comparison. Different superscripts differ at *p* < 0.05. Orthogonal polynomial contrasts analysis was conducted to see linear pattern (L) and quadratic pattern (Q) among the treatments. To calculate the optimal dosage of tannic acid in broilers, segmented linear regression was conducted, and *X0* (inflection point) with *p* values for slope 1 (lower than *X0*) and slope 2 (higher X0) were presented.

**FIGURE 15:**
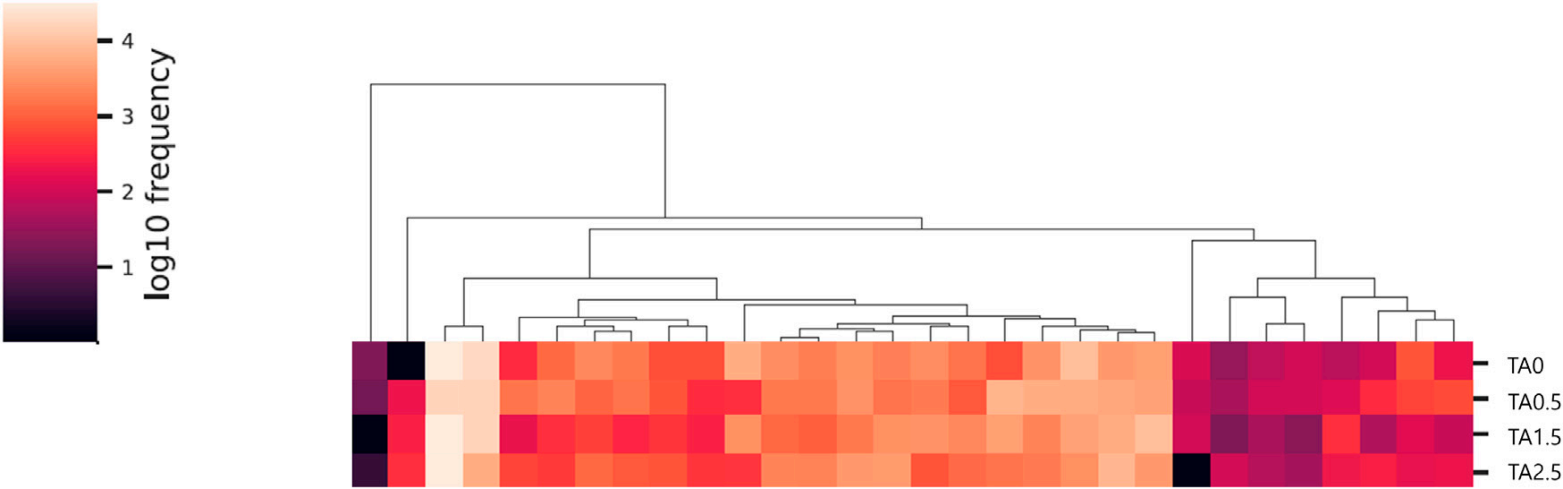
Heatmap of cecal microbiome in the broilers of the tannic acid 0 (TA0): basal diet without tannic acid; 2) tannic acid 0.5 (TA0.5): basal diet with 0.5 g/kg tannic acid; 3) tannic acid 1.5 (TA1.5); and 4) tannic acid 2.5 (TA2.5) groups on D 21.

The phylum level composition of the cecal microbial communities is shown in Figure 13. The relative abundance of the phylum Bacteroidetes tended to be increased by supplemental TA (*p* = 0.1), whereas the relative abundance of the phylum proteobacteria tended to be decreased by supplemental TA (*p* = 0.1). Family level composition of the cecal microbial communities is presented in Figure 14. The relative abundance of the family Rikenellaceae tended to be linearly reduced by TA at levels less than 500 mg/kg (*p* = 0.05) and tended to be linearly increased at levels greater than 500 mg/kg (*p* = 0.1). The relative abundance of unclassified Clostridiales was linearly decreased (*p* < 0.05) by supplemental TA. The relative abundance of the family Bacillaceae was quadratically modulated (*p* < 0.05) and linearly reduced at levels less than 1,045 mg/kg (*p* < 0.05) and tended to be linearly increased at levels greater than 1,045 mg/kg (*p* = 0.06). The heatmap visually presents that there were differences at all levels in the composition of cecal microbial communities by supplemental TA (Figure 15).

### Correlation between the cecal microbial composition with volatile fatty acid production and parameters of growth performance, fat accumulation, bone health, fat metabolism mRNA expression, apparent ileal digestibility, immune system, gut barrier integrity, brush border digestive enzymes, nutrient transporters, antioxidant capacity, and volatile fatty acid production

The cecal microbial composition and VFA were positively or negatively correlated with diverse parameters of growth performance, fat accumulation, bone health, fat metabolism mRNA expression, AID, brush border digestive enzyme activities, nutrient transporters, immune system, gut barrier integrity, antioxidant capacity, and VFA production (Table 3; *p* < 0.05). To be specific, the phylum Bacteroidetes was negatively correlated with body fat percentage (*p* < 0.05; *R* ^2^ = −0.368) and SOD activities (*p* < 0.05; *R* ^2^ = −0.411). The phylum Firmicutes were positively correlated with EAAT3 (*p* < 0.05; *R* ^2^ = −0.358), SGLT1 (*p* < 0.05; *R* ^2^ = −0.429), and JAM2 (*p* < 0.05; *R* ^2^ = −0.36). The order Clostridiales were positively correlated with PepT1 (*p* < 0.05; *R* ^2^ = 0.366), MUC2 (*p* < 0.05; *R* ^2^ = 0.365), and NFκB (*p* < 0.05; *R* ^2^ = 0.365). The family Bacillaceae was positively correlated with jejunal lipase activities (*p* < 0.05; *R* ^2^ = 0.358), cecal acetate production (*p* < 0.05; *R* ^2^ = 0.504), cecal VFA production (*p* < 0.05; *R* ^2^ = 0.457) but negatively correlated with AID of CP (*p* < 0.05; *R* ^2^ = 0.358). The cecal acetate concentration was positively correlated with jejunal sucrase activities (*p* < 0.05; *R* ^2^ = 0.428), jejunal APN activities (*p* < 0.05; *R* ^2^ = 0.43), jejunal IAP activities (*p* < 0.05; *R* ^2^ = 0.406) but negatively correlated with AID of DM (*p* < 0.05; *R* ^2^ = −0.368) and AID of OM (*p* < 0.05; *R* ^2^ = −0.371). Total VFA was positively correlated with jejunal sucrase activities (*p* < 0.05; *R* ^2^ = 0.395), jejunal APN activities (*p* < 0.05; *R* ^2^ = 0.414), jejunal IAP activities (*p* < 0.05; *R* ^2^ = 0.401) but negatively correlated with liver TAC (*p* < 0.05; *R* ^2^ = −0.364), AID of DM (*p* < 0.05; *R* ^2^ = −0.369) and AID of OM (*p* < 0.05; *R* ^2^ = −0.37).

**TABLE 3:** Correlation between the cecal microbial composition with volatile fatty acids and growth performance (ADFI), fat accumulation (fat %), bone health (BMC and BMD), fat metabolism mRNA expression (ACOX1, APOB, CPT1A, FABP2, FABP4, FASN, and PPARγ), immune system (IL1β), gut barrier integrity (JAM2 and MUC2), brush border digestive enzymes (APN, Lipase, Sucrase, Maltase, and IAP), antioxidant capacity (TAC and SOD), apparent ileal digestibility (DM, OM, and CP), nutrient transporters (EAAT3, B0AT1, SGLT1, and PepT1), and volatile fatty acid production parameters (acetate, propionate, and isobutyrate) with significant differences (*p* < 0.05) in broilers fed different concentration of tannic acid on D 21.

	Items	*p* value	*R* ^2^	Items	*p* value	*R* ^2^	Items	*p* value	*R* ^2^	Items	*p* value	*R* ^2^
Bacteroidetes	Fat %	0.042	−0.368	CPT1A	0.023	0.401	SOD	0.022	−0.411			
Actinobacteria	FABP2	0.012	0.440									
Proteobacteria	Lipase	0.042	0.367									
Firmicutes	EAAT3	0.048	0.358	SGLT1	0.016	0.429	JAM2	0.047	0.360			
Actinobacteria	IL1β	0.019	0.420	Iso butyrate	0.046	−0.361						
Tenericutes	IL1β	0.037	0.377									
Enterobacteriaceae	Lipase	0.026	0.399									
Bacteroidaceae	ADFI	0.044	−0.364	ACOX1	0.007	0.470	CPT1A	0.003	0.507	PPARγ	0.003	0.506
	Propionate	0.017	0.426									
Lachnospiraceae	BMD	0.019	0.420	BMC	0.048	0.358	Valerate	0.013	−0.440			
Clostridiales; f__	PepT1	0.043	0.366	MUC2	0.044	0.365	NFκB	0.048	0.358			
Ruminococcaceae	IAP	0.014	0.439	NFκB	0.042	−0.368						
Lactobacillaceae	ADFI	0.003	0.513	Lipase	0.016	0.430	NFκB	0.028	−0.394	SOD	0.006	−0.513
	Ash	0.006	0.481									
Bacillaceae	PPARγ	0.020	0.409	Lipase	0.048	0.358	CP	0.049	−0.357	Acetate	0.004	0.504
	Total VFA	0.010	0.457									
Coriobacteriaceae	FABP2	0.009	0.456	FABP4	0.047	0.354	IL1β	0.024	0.405	Iso butyrate	0.041	−0.369
Clostridiales; __	B0AT1	0.001	0.549	EAAT3	0.016	0.428	SGLT1	0.017	0.425	NFκB	0.011	0.449
Clostridiaceae	PepT1	0.012	0.446	MUC2	0.032	0.386	IL1β	0.005	0.490	DM	0.002	−0.541
	OM	0.002	−0.535	Ash	0.010	−0.457	CP	0.006	−0.484			
Erysipelotrichaceae	ACOX1	0.001	0.542	APOB	0.003	0.507	FABP4	0.004	0.491	FASN	0.003	0.51
Acetate	Sucrase	0.016	0.428	APN	0.016	0.430	IAP	0.024	0.406	DM	0.042	−0.368
	OM	0.040	−0.371									
Propionate	CPT1A	0.002	0.531	PPARγ	0.024	0.398	GSSG	0.026	0.400	Ash	0.010	−0.454
Isobutyrate	Maltase	0.032	0.385									
Butyrate	TAC	0.018	−0.422									
Isovalerate	Fat %	0.003	−0.517	Fat weight	0.021	−0.413	DM	0.014	−0.438	OM	0.017	−0.424
	Ash	0.005	−0.495	CP	0.023	−0.406						
Valerate	PPARγ	0.046	0.355	Ash	0.041	−0.370						
Total VFA	Sucrase	0.028	0.395	APN	0.021	0.414	IAP	0.025	0.401	TAC	0.044	−0.364
	DM	0.041	−0.369	OM	0.041	−0.370						

## Discussion

The purposes of the study were to investigate the effects of supplemental TA on growth performance, gut health, antioxidant status, bone mineral density, body composition, and cecal VFA concentrations and microbiome in broilers and to calculate the optimal concentration of supplemental TA in broilers based on the parameters. In the current study, BW were linearly reduced as supplemental TA levels increased in the three phases (D 7, 14, and 21). Reduced BW in broiler fed supplemental TA on D 21 would be primarily due to reduced feed intake on D 7 to 14 because no statistical differences were observed in ADG, ADFI and FCR on D 21 in the current study. High concentrations of TA can reduce feed intake in broilers by causing irritation in esophagus and necrosis in crop, gizzard, and duodenum (Suleyman, 2017). The BW of broilers on D 21 started to be decreased when greater than 972 mg/kg of supplemental TA was offered to the birds, which implies that TA at levels less than 972 mg/kg can be used in broilers without compromising growth rate in broilers.

In the current study, AID of CP and EE were reduced by supplemental TA. Tannins are considered as anti-nutritional factors mainly because TA interacts with dietary and endogenous proteins (e.g., digestive enzymes), which impairs nutrient digestion and absorption in broilers (Ortiz et al., 1993). Because there were no statistical differences in the jejunal morphology, and APN activities and amino acid and peptide transporters (e.g., B0AT1, EAAT3, and PepT1) in the present study, interaction of TA with dietary proteins might have been the main factor that reduced AIP of CP in broilers. In the current study, AID of EE was decreased by supplemental TA in broilers along with reduced AID of CP. Reduced AID of EE might be closely associated with reduced lipase activities in the current study. The TA potentially impaired activities of lipase by forming a complex with lipase (Moreno-Córdova et al., 2020). Moreover, TA may have interacted with dietary lipid sources and bile salts. Fan et al. (2013) showed that TA inhibited the production of lipid droplets, which can be an obstacle to be digested by the host. A previous study by Li et al. (2019) reported that condensed tannins can precipitate bile salts that play an important role in facilitating lipid digestions. Along with decreased AID of CP and EE and mRNA expression of the glucose transporter (SGLT1), supplemental TA possibly reduced the AID of gross energy in the current study. Reduce AID of nutrients may have negatively affected growth performance in broilers in the current study.

Intestinal mucus, which is produced by goblet cells, play important roles in facilitating nutrient digestion and absorption, protecting the enterocytes, and preventing invasion of pathogens (Duangnumsawang et al., 2021). Goblet cells are derived from multipotent stem cells near the base of the crypts, and immature goblet cells starts to rapidly synthesize and secret mucus (Kim and Khan, 2013). In the current study, supplemental TA linearly reduced goblet cells in the crypts of the jejunum in broilers. This may have been closely associated with reduced AID of CP because certain amino acids (threonine, serine, and proline) are required to produce mucus efficiently (Koo et al., 2020). Deficiency of these amino acids may have disturbed goblet cell proliferation and mucus production. Consequently, reduced mucus production by supplemental TA potentially impaired nutrient digestion and absorption. Sicard et al. (2017) reported that pathogenic bacteria need intestinal mucus for their colonization. However, it is unclear whether reduced mucus production by supplemental TA at levels greater than 784.9 mg/kg can be beneficial for broilers infected with pathogens (e.g., *Clostridium perfringens*) because some pathogens can use mucus for colonization. Thus, further studies are necessary to understand mucus production and pathogen colonization in broilers under bacterial infection conditions.

The tight junction proteins, such as JAM2 and ZO2, play an important role in maintaining gut barrier integrity (Suzuki, 2020). In the current study, the relative mRNA expression of ZO2 was reduced by supplemental TA, which suggests that supplemental TA can impair gut barrier integrity in broilers. Moreover, relative mRNA expression of MUC2, which is associated with gut barrier integrity, was reduced in this study. In contrast, many studies showed that supplemental TA improved gut barrier integrity in pigs (Yu et al., 2020; Song et al., 2021) and mice (Scaldaferri et al., 2014) in challenging conditions (e.g., weaning and intestinal bowel diseases) potentially *via* showing antimicrobial, anti-inflammatory and antioxidative effects. Although the current study showed that supplemental TA impaired gut barrier integrity in broilers without any challenging conditions, it is unknown how TA supplementation would affect gut barrier integrity in broilers under challenge conditions. Therefore, more future studies are required to investigate the beneficial effects of supplemental TA on gut barrier integrity in broilers in challenge conditions including pathogenic infection, heat stress, etc.

A previous study by Marzo et al. (2002) reported that high concentrations of dietary TA supplementation (25 g/kg) impaired liver functions in broilers by showing proteolytic activities. Our current study showed that supplementation of TA (0–2.5 g/kg) linearly increased GSSG and decreased the ratio of reduced GSH and GSSG, which implies that supplemental TA may induced oxidative stress in the liver. Although TA is known to have strong antioxidative capacity in *vitro* conditions (Kim et al., 2010), TA can cause oxidative stress in the liver by exhibiting proteolytic activities. However, the TA0.5 group had the highest activities of SOD, an endogenous enzymatic antioxidant (Albarran et al., 2001), among the treatments in the present study. Therefore, supplementation of TA can modulate endogenous antioxidant capacity in broilers.

In the current study, the modulation of microbiota and their activities by supplemental TA reduced concentrations of acetate, butyrate, valerate, and total VFA in the cecal contents of broilers. The VFA are important microbial metabolites and energy sources for gut health and growth in broilers (Choi et al., 2021); thus poor production of VFA can result in a reduction in growth performance and fat accumulation (Fluitman et al., 2017). The present study showed that concentrations of propionate and valerate were positively correlated to CPT1A (*p* < 0.05; *R* ^2^ = 0.531) and PPARγ (*p* < 0.05; *R* ^2^ = 0.355), respectively. This suggests that VFA production can potentially affect fat metabolism in chickens fed TA. In addition, VFA are also important energy sources for the development of enterocytes in the intestine of broilers (Bergman, 1990). In the current study, total VFA, acetate, and isobutyrate were positively correlated with brush border digestive enzymes (sucrase, APN, and IAP) which implies that VFA are important to induce gut development in chickens fed TA.

In the present study, supplemental TA tended to linearly reduce alpha diversity indices (diversity within a sample) including species equitability, expressed as pielou evenness, and faith phylogenetic diversity index in the cecal microbial communities of broilers. As the name refers, the pielou evenness was for measuring the evenness of microbial communities, and faith phylogenetic diversity was for measuring biodiversity based on phylogeny (the tree of life) (González-Mercado et al., 2020). While it is still controversial, lower alpha diversity in cecal microbial communities may indicate less stable and undeveloped microbial community compared to the higher alpha diversity (Choi et al., 2015). However, a previous study by Diaz (Diaz Carrasco et al., 2018) reported that 1 g/kg supplemental TA increased shannon’s diversity in broilers on D 30. The difference would be originated from different tannins sources [blend of tannins derived from chestnut (*Castanea sativa*) and quebracho (*Schinopsis lorentzii*; condensed tannins)] and different age of the broilers. No differences were observed in the beta diversity (diversity among treatments) in the current study.

In the present study, supplemental TA increased the relative abundance of the phylum Bacteroidetes in broilers. The phylum Firmicutes and the phylum Bacteroidetes are the main bacterial groups in the ceca of broilers (Wang et al., 2021a), and their relative abundance and ratio are closely associated with fat accumulation and nutrient absorption (Singh et al., 2013). Our current study showed that the relative abundance of the phylum Bacteroidetes was negatively correlated with body fat percentage (*p* < 0.05, *R* ^2^ = −0.368), and the relative abundance of the phylum Firmicutes were positively correlated with EAAT3 (*p* < 0.05, *R* ^2^ = 0.538) and SGLT1 (*p* < 0.05, *R* ^2^ = 0.429) in broilers fed TA. Moreover, the phylum Bacteroidetes were negatively correlated with the activities of SOD (*p* < 0.05, *R* ^2^ = −0.411), and the phylum Firmicutes were positively correlated with mRNA expression of JAM2 (*p* < 0.05, *R* ^2^ = 0.360) in broilers fed TA. This implies that supplemental TA negatively affected microbiota of broilers for better growth performance and fat accumulation. However, the relative abundance of the phylum Proteobacteria, which includes many pathogenic genera (*Escherichia*, *Salmonella*, *Vibrio*, etc) (Ma et al., 2017), were reduced by supplemental TA. Reduced relative abundance of the phylum Proteobacteria may indicate better intestinal health and less pathogenic bacteria in broilers (Prasai et al., 2016). This would be due to strong antimicrobial effects of TA against certain pathogens such as *Salmonella* spp., *Escherichia coli* (Kim et al., 2010). Reduced the relative abundance of the phylum Proteobacteria may be closely associated with decreased relative mRNA expression of IL2 and IL6, proinflammatory cytokines, which indicates that TA supplementation potentially reduces inflammation by exhibiting antimicrobial effects in broilers. Based on antimicrobial and anti-inflammatory effects of TA, TA supplementation can be beneficial in broilers infected with pathogens in antibiotic-free conditions.

Pham et al. (2020) reported that the relative abundance of the family Rikenellaceae has been positively correlated with gastrointestinal tract dysfunctions. In the current study, the relative abundance of the family Rikenellaceae was decreased by dietary TA at levels less than 500 mg/kg and increased at levels greater than 500 mg/kg, which indicates that 500 mg/kg supplemental TA can be beneficial for gut health, whereas higher than 500 mg/kg supplemental TA can be detrimental for gut health of broilers. Supplemental TA reduced relative abundance of the unclassified Clostridiales in the current study, and its relative abundance was positively correlated with PepT1 (*p* < 0.05; *R* ^2^ = 0.546), MUC2 (*p* < 0.05; *R* ^2^ = 0.365), and NFκB (*p* < 0.05; *R* ^2^ = 0.358). Torok et al. (2011) also showed that the order Clostridiales are known to be positively correlated with growth performance. Our current study showed that the relative abundance of the family Bacillaceae peaked at 1,045 mg/kg supplemental TA. The relative abundance of the family Bacillaceae were positively correlated with acetate (main VFA) (*p* < 0.05; *R* ^2^ = 0.504), and total VFA (*p* < 0.05; *R* ^2^ = 0.457). In agreement, Wasti et al. (2021) reported that the relative abundance of the family Bacillaceae was positively correlated with growth performance and VFA production. Based on the family level composition of cecal microbial communities, approximately 500–1,000 mg/kg of TA may promote a better gut microbial profile and VFA production.

In this study, bone mineral density and bone mineral content were reduced by supplemental TA, which indicates that supplemental TA reduced bone health in broilers. This result is in agreement with previous studies which reported that tannins can negatively affect bone health of broilers (Abbel-Monein, 2013; Tomaszewska et al., 2018). Tannins are known to inhibit the utilization of calcium, phosphorus, and iron by precipitation, which can negatively affect bone development in animals (Hassan et al., 2003). However, there were no differences in AID of ash in the present study. More studies are required to investigate the direct effects of supplemental TA on bone development and maintenance in broilers.

In the current study, the fat accumulation and body fat percentage were reduced, and lean to fat ratio was increased by supplemental TA in broilers. These results indicate that the decrease in the fat accumulation was more pronounced compared to the reduction level in lean accumulation by supplemental TA in broilers. Reduced fat accumulation can be closely associated with reduced AID of EE, decreased activities of lipase, and modulated microbiota with VFA production in the current study. Liver was selected for fat metabolism analyses because liver is the main area for fat synthesis and metabolism in chickens (Zaefarian et al., 2019). However, mRNA expression of genes related to fat metabolism in the liver was not modulated by supplemental TA in the current study. Many studies have reported that TA modulated fat metabolism in the liver of the animals (Park et al., 2002; Huang et al., 2013; Huang et al., 2019). Potentially, different alteration of lipid digestibility, VFA production, and microbiome by supplemental TA may have different impacts on the mRNA expression of genes associated with fat metabolism when the current study was compared to other studies. Many previous studies showed that dietary TA exhibit antinutritional effects mainly by interacting with dietary and endogenous proteins in broilers (Redondo et al., 2014). In contrast, our current study also suggests that dietary TA had more severe effects on the fat accumulation rather than protein accumulation in broilers.

**FIGURE 16:**
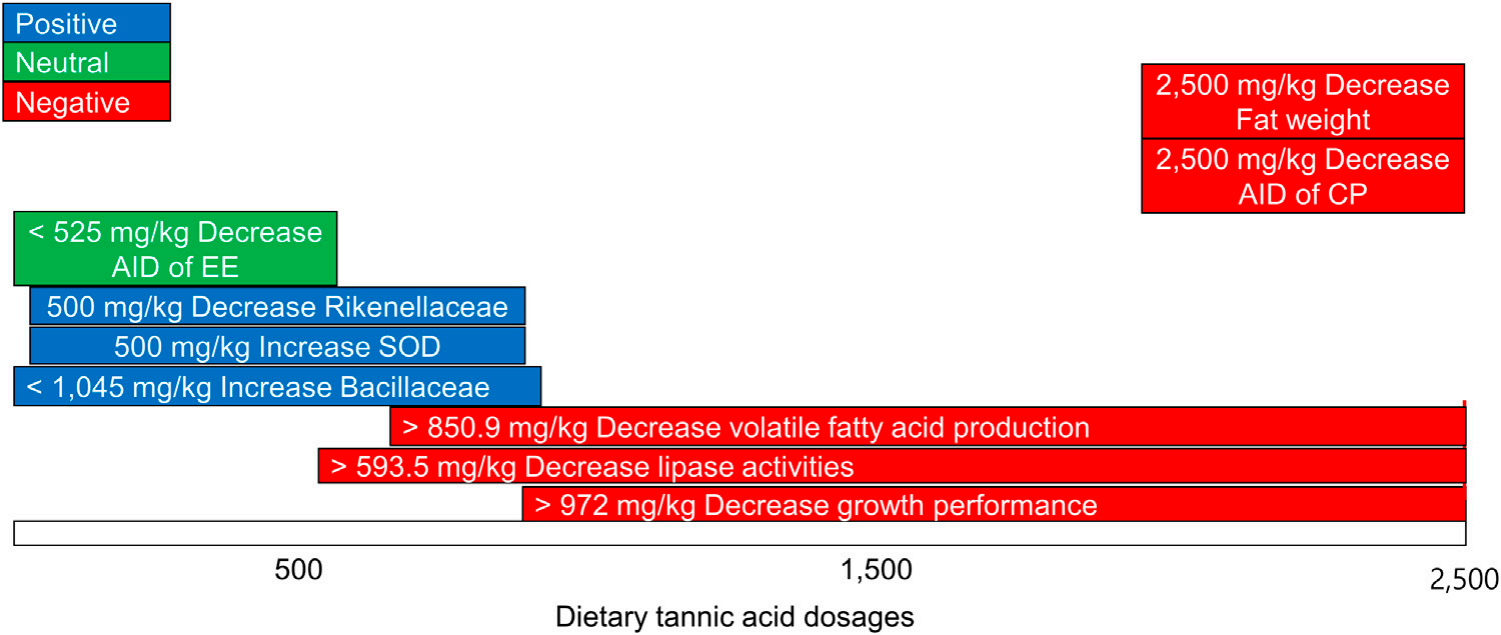
Summary of different effects of diverse doses of dietary tannic acid in broilers. SOD, superoxide dismutase; AID, apparent ileal digestibility, EE, ether extracts; CP, crude protein.

To sum up, supplementation of TA (0–2.5 g/kg) reduced growth performance, decreased nutrient utilization, increased lean to fat ratio, and negatively modulated microbiota in broilers. However, supplemental TA up to certain levels did not negatively influence growth performance at levels less than 972 mg/kg, lipase activities at levels less than 593.5 mg/kg, total VFA production at levels less than 850.9 mg/kg, and cecal microbiota at levels less than 1,045 mg/kg in broilers (Figure 16). Furthermore, 500 mg/kg of supplemental TA increased activities of SOD in the liver and decreased the relative abundance of the family Rikenellaceae. Therefore, approximately supplementation of 500–1,000 mg/kg TA have potentials to show beneficial effects on growth performance and gut health in broilers under antibiotic-free conditions.

## Data Availability

The data presented in the study are deposited in the NCBI repository, accession number PRJNA852339.
